# Type II alveolar epithelial cell–specific loss of RhoA exacerbates allergic airway inflammation through SLC26A4

**DOI:** 10.1172/jci.insight.148147

**Published:** 2021-07-22

**Authors:** Danh C. Do, Yan Zhang, Wei Tu, Xinyue Hu, Xiaojun Xiao, Jingsi Chen, Haiping Hao, Zhigang Liu, Jing Li, Shau-Ku Huang, Mei Wan, Peisong Gao

**Affiliations:** 1 Division of Allergy and Clinical Immunology, Johns Hopkins University School of Medicine, Baltimore, Maryland, USA.; 2 Department of Respiratory Medicine, Xiangya Hospital, Central South University, Changsha, Hunan, China.; 3 Department of Respirology & Allergy, Third Affiliated Hospital of Shenzhen University, Shenzhen, China.; 4 Institute of Allergy and Immunology, School of Medicine, Shenzhen University, Shenzhen, China.; 5 Children’s Hospital, Chongqing Medical University, Chongqing, China.; 6 JHMI Deep Sequencing and Microarray Core Facility, Johns Hopkins University School of Medicine, Baltimore, Maryland, USA.; 7 Department of Allergy and Clinical Immunology, Guangzhou Institute of Respiratory Health, State Key Laboratory of Respiratory Disease, The First Affiliated Hospital of Guangzhou Medical University, Guangzhou, China.; 8 National Institute of Environmental Health Sciences, National Health Research Institutes, Miaoli, Taiwan.; 9 Department of Orthopaedic Surgery, Johns Hopkins University School of Medicine, Baltimore, Maryland, USA.

**Keywords:** Immunology, Inflammation, Allergy, Asthma, Th2 response

## Abstract

The small GTPase RhoA and its downstream effectors are critical regulators in the pathophysiological processes of asthma. The underlying mechanism, however, remains undetermined. Here, we generated an asthma mouse model with RhoA–conditional KO mice (*Sftpc-cre;RhoA^fl/fl^*) in type II alveolar epithelial cells (AT2) and demonstrated that AT2 cell–specific deletion of RhoA leads to exacerbation of allergen-induced airway hyperresponsiveness and airway inflammation with elevated Th2 cytokines in bronchoalveolar lavage fluid (BALF). Notably, *Sftpc-cre;RhoA^fl/fl^* mice showed a significant reduction in Tgf-β1 levels in BALF and lung tissues, and administration of recombinant Tgf-β1 to the mice rescued Tgf-β1 and alleviated the increased allergic airway inflammation observed in *Sftpc-cre;RhoA^fl/fl^* mice. Using RNA sequencing technology, we identified Slc26a4 (pendrin), a transmembrane anion exchange, as the most upregulated gene in RhoA-deficient AT2 cells. The upregulation of SLC26A4 was further confirmed in AT2 cells of asthmatic patients and mouse models and in human airway epithelial cells expressing dominant-negative RHOA (RHOA-N19). SLA26A4 was also elevated in serum from asthmatic patients and negatively associated with the percentage of forced expiratory volume in 1 second (FEV_1_%). Furthermore, SLC26A4 inhibition promoted epithelial TGF-β1 release and attenuated allergic airway inflammation. Our study reveals a RhoA/SLC26A4 axis in AT2 cells that functions as a protective mechanism against allergic airway inflammation.

## Graphical abstract

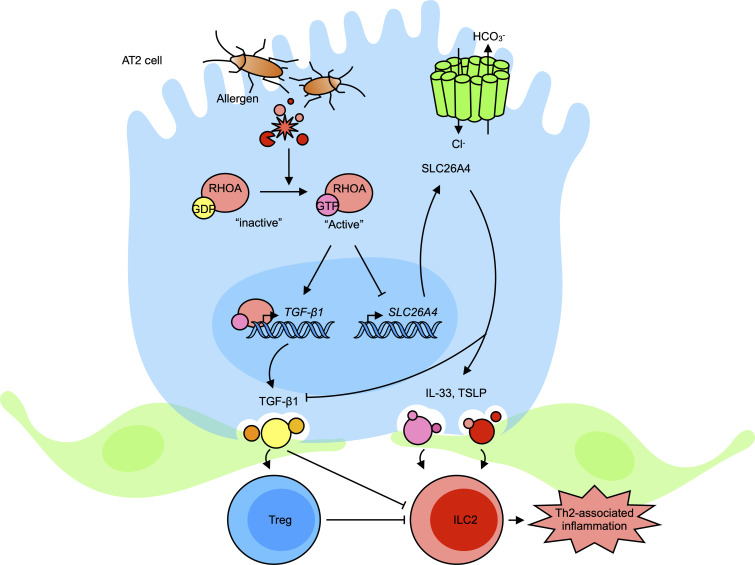

## Introduction

Asthma is a serious chronic inflammatory disease, with an increasing prevalence worldwide (1, 2). Environmental allergen exposure (e.g., cockroach, house dust mite [HDM]) has been considered to be one of the major risk factors for asthma (3). Th2-associated inflammation with elevated Th2 cytokines represents the typical adaptive immune response to allergen exposure in patients with allergic asthma (4). Th2 cytokines, IL-4, IL-5, and IL-13, can induce the recruitment of eosinophils to the airway and promote airway hyperresponsiveness (AHR) (5–7). Airway epithelial cells are the first line of defense against environmental allergens (8) and secrete IL-33, IL-25, and thymic stromal lymphopoietin (TSLP) that can induce Th2 cells and type 2 innate lymphoid cell (ILC2) activation to potentiate allergen-induced Th2-associated airway inflammation (9, 10). However, the biological mechanisms underlying the allergen exposure–induced epithelial cell activation and subsequent downstream Th2-associated airway inflammation in asthma remain unclear.

The ras homolog family member A (RhoA) of the Rho family GTPases is an intracellular signal transducer. It is a nucleotide-dependent protein, switching between an inactive form, which is GDP-bound, and an active form, GTP-bound RhoA (11). Upregulated expression and activation of RhoA have been associated with bronchial smooth muscle contraction, AHR, and airway remodeling in asthma (12–18). Of interest, a feed-forward connection between RhoA signaling and TGF-β1 has been suggested to drive airway constriction and AHR in asthma (19–21). Recent advances have suggested that RhoA also plays a critical role in airway inflammation through affecting recruitment, differentiation, and activation of various inflammatory cells (e.g., eosinophils, macrophages, mast cells, and T cells) (14, 22, 23). Disruption of RhoA in T cells has been shown to inhibit cell activation and Th2 differentiation and to prevent allergen-induced lung inflammation by affecting glycolysis and oxidative phosphorylation (22). Furthermore, the disruption of RhoA in T cells can also suppress Th17 responses and neutrophil-involved airway inflammation in an allergen-induced asthma model (14). We have recently reported increased activation of RhoA signaling in asthmatic airway epithelial cells (23). Of note, type II alveolar epithelial cells (AT2) have a unique function in producing surfactant and serve as progenitor cells in response to lung injury (24, 25). AT2 cell damage has been implicated in lung fibrosis (26) and inflammation (27). However, the genetic regulation and immune function of AT2 cell function in the development of allergic airway inflammation and asthma remain unknown.

Here, we found that RhoA in AT2 cells plays a critical role in regulating allergen-induced AHR and Th2-associated airway inflammation in allergen-induced mouse models of asthma. One of the striking findings is that the mice with AT2 cell–specific RhoA deficiency (*Sftpc-cre;RhoA^fl/fl^*) showed a significant reduction in Tgf-β1 levels in BALF and lung tissues, and treatment with recombinant Tgf-β1 (rTgf-β1)reversed the increased airway inflammation observed in *Sftpc-cre;RhoA^fl/fl^* mice. Most importantly, RNA sequencing (RNA-seq) analysis identified Slc26a4, a transcript involved in Cl^–^ conductance, to be one of the most upregulated genes in RhoA-deficient AT2 cells. Further studies demonstrated that SLC26A4 is increased in AT2 cells and serum of asthmatic patients and is negatively associated with forced expiratory volume in 1 second (FEV_1_) among asthmatics. Inhibition of the airway chloride channel led to an increased release of TGF-β1 but suppression of allergic airway information, suggesting that SLA26A4 may represent a therapeutic target to tackle asthma.

## Results

### AT2 cell–specific deletion of RhoA exacerbates the CRE-induced AHR and inflammation.

**Figure 1:**
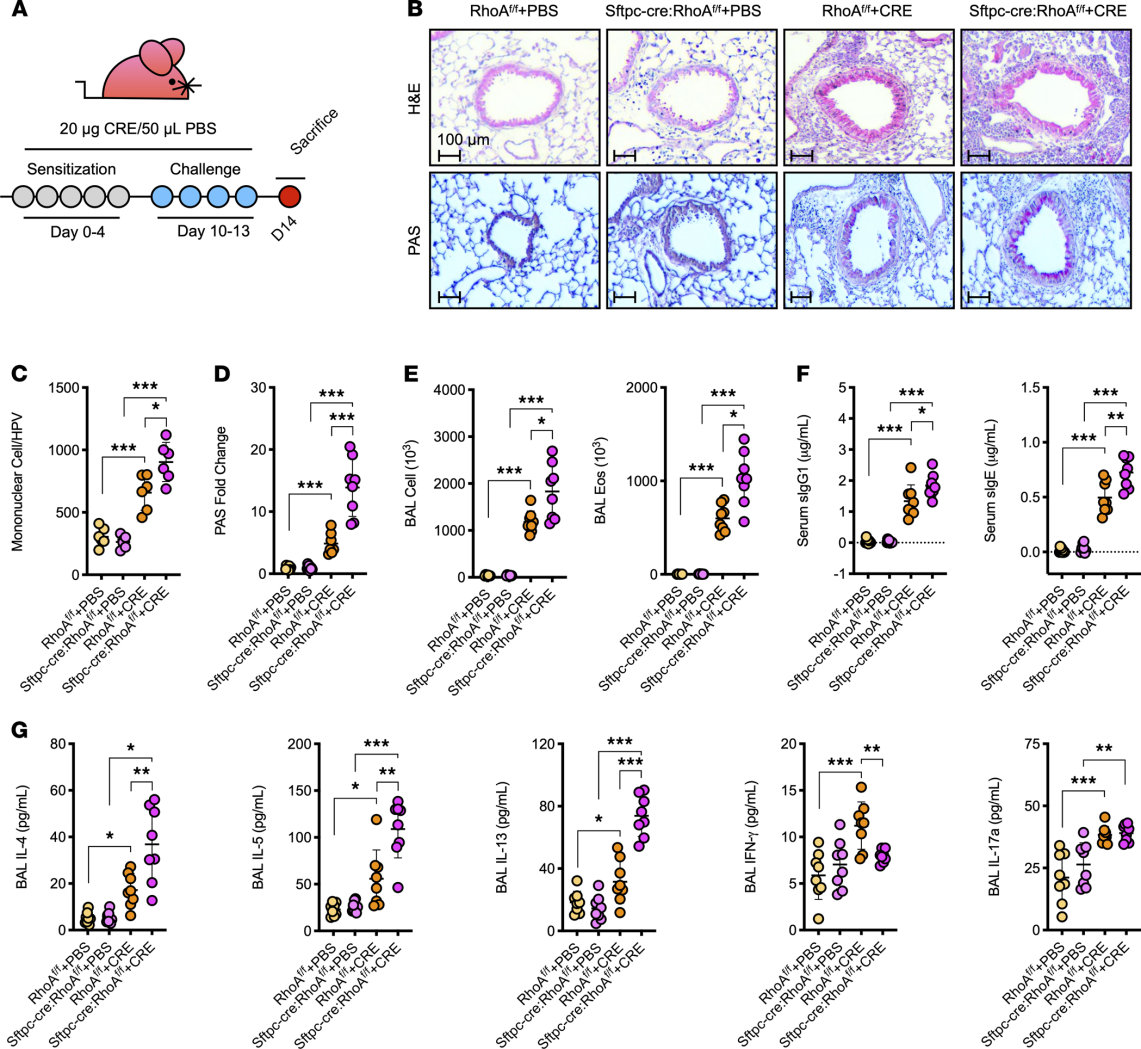
Deletion of RhoA in AT2 RhoA leads to the exacerbation of CRE-induced (CRE-induced) airway inflammation. (**A**) Protocol for CRE-induced mouse model of asthma. (**B**) Representative lung sections stained with H&E (upper panel) and Periodic acid–Schiff (PAS, lower panel). (**C**) Quantification of mononuclear cell infiltrates in H&E-stained lung sections (*n* = 6). (**D**) Goblet cells quantification in PAS-stained lung sections (*n* = 7–9). (**E**) BALF total and eosinophil cell counts as determined by flow cytometry (*n* = 8). (**F**) Serum levels of CRE-specific IgE and IgG1 (*n* = 8). (**G**) BALF levels of cytokines (*n* = 8). Data represent mean ± SEM of 2 independent experiments. Group comparisons were made using 2-way ANOVA. *P < 0.05, **P < 0.01, and ***P < 0.001. Scale bars: 100 μm.

Our previous studies found an increased expression of active RhoA in airways from patients with asthma (23), but its role in airway epithelial cells remains unexplored. To investigate the functional significance of RhoA upregulation in the airway epithelium in asthma, we deleted RhoA from type II alveolar airway epithelium (AT2) by crossing a floxed RhoA mouse (*RhoA^fl/fl^*) with a *Sftpc-cre* mouse (Supplemental Figure 1A; supplemental material available online with this article; https://doi.org/10.1172/jci.insight.148147DS1). The mice were confirmed by genotyping (Supplemental Figure 1B). The deletion of RhoA in AT2 cells was confirmed by coimmunostaining with RhoA and Sfptc, a marker for AT2 cells, in the lung tissues (Supplemental Figure 1C). In addition to AT2 cells, Sfptc expression was also found in basal cells but not in type I alveolar airway epithelium (AT1) and central airways (Supplemental Figure 1D). Further analysis confirmed that *Sftpc-cre;RhoA^fl/fl^* mice had the deletion of RhoA in AT2 cells, but not in the central airways and basal cells of lung tissues (Supplemental Figure 1, E and F). The mice were then subjected to our established CRE (CRE) asthma model as illustrated in Figure 1A. Compared with WT mice, CRE-treated *Sftpc-cre;RhoA^fl/fl^* mice showed a significant increase in airway resistance (Supplemental Figure 2). Furthermore, the AT2 cell–specific RhoA deletion led to the exacerbation of allergic lung inflammation. Particularly, *Sftpc-cre;RhoA^fl/fl^* mice showed a significant increase in the recruitment of inflammatory cells to the lung with dense peribronchial infiltrates (Figure 1, B and C; H&E) and goblet cell hyperplasia (Figure 1, B and D; PAS) in the histological examination. The total inflammatory cells, especially eosinophils, were markedly increased in the bronchoalveolar lavage fluids (BALF) of *Sftpc-cre;RhoA^fl/fl^* mice (Figure 1E). No difference was observed for neutrophils in the BALF (Supplemental Figure 3A). Furthermore, *Sftpc-cre;RhoA^fl/fl^* mice showed higher levels of CRE-specific IgE and IgG1 in serum (Figure 1F). These mice also showed elevated IL-4, IL-5, and IL-13 but reduced IFN-γ in BALF (Figure 1G). No clear change was observed for IL-17. Together, these data suggest that A2 RhoA protects against CRE-induced AHR and Th2-associated airway inflammation.

### AT2 cell–specific deletion of RhoA exacerbates the HDM-induced airway inflammation.

To determine whether the CRE-induced increased airway inflammation in *Sftpc-cre;RhoA^fl/fl^* mice could be applied to other allergens, we utilized another clinically relevant allergen, HDM, to generate a mouse asthma model, as illustrated in Supplemental Figure 4A. Similar to the CRE model, loss of RhoA in AT2 cells led to an increase in HDM-induced airway inflammation. In particular, histological analysis of lung tissues from *Sftpc-cre;RhoA^fl/fl^* mice demonstrated an increase in the recruitment of inflammatory cells to the lung with dense peribronchial infiltrates (Supplemental Figure 4, B and C; H&E) and goblet cell hyperplasia (Supplemental Figure 4, B and D; PAS). HDM-treated *Sftpc-cre;RhoA^fl/fl^* mice also showed a higher number of total inflammatory cells — especially eosinophils (Supplemental Figure 4E) but not neutrophils (Supplemental Figure 3B) in BALF — and higher levels of HDM-specific IgE and IgG1 in serum (Supplemental Figure 4F). Furthermore, compared with WT mice, *Sftpc-cre;RhoA^fl/fl^* mice showed increased levels of IL-4, IL-5, and IL-13 in BALF (Supplemental Figure 4G). In contrast, these mice showed a reduction in Th1 cytokine IFN-γ, but no change was noted for Th17 cytokine IL-17 in BALF. These results support that AT2 RhoA prevents not only CRE-induced, but also HDM allergen-induced airway inflammation.

### AT2 cell–specific deletion of RhoA leads to changes in cytokine release of asthma mouse models.

**Figure 2:**
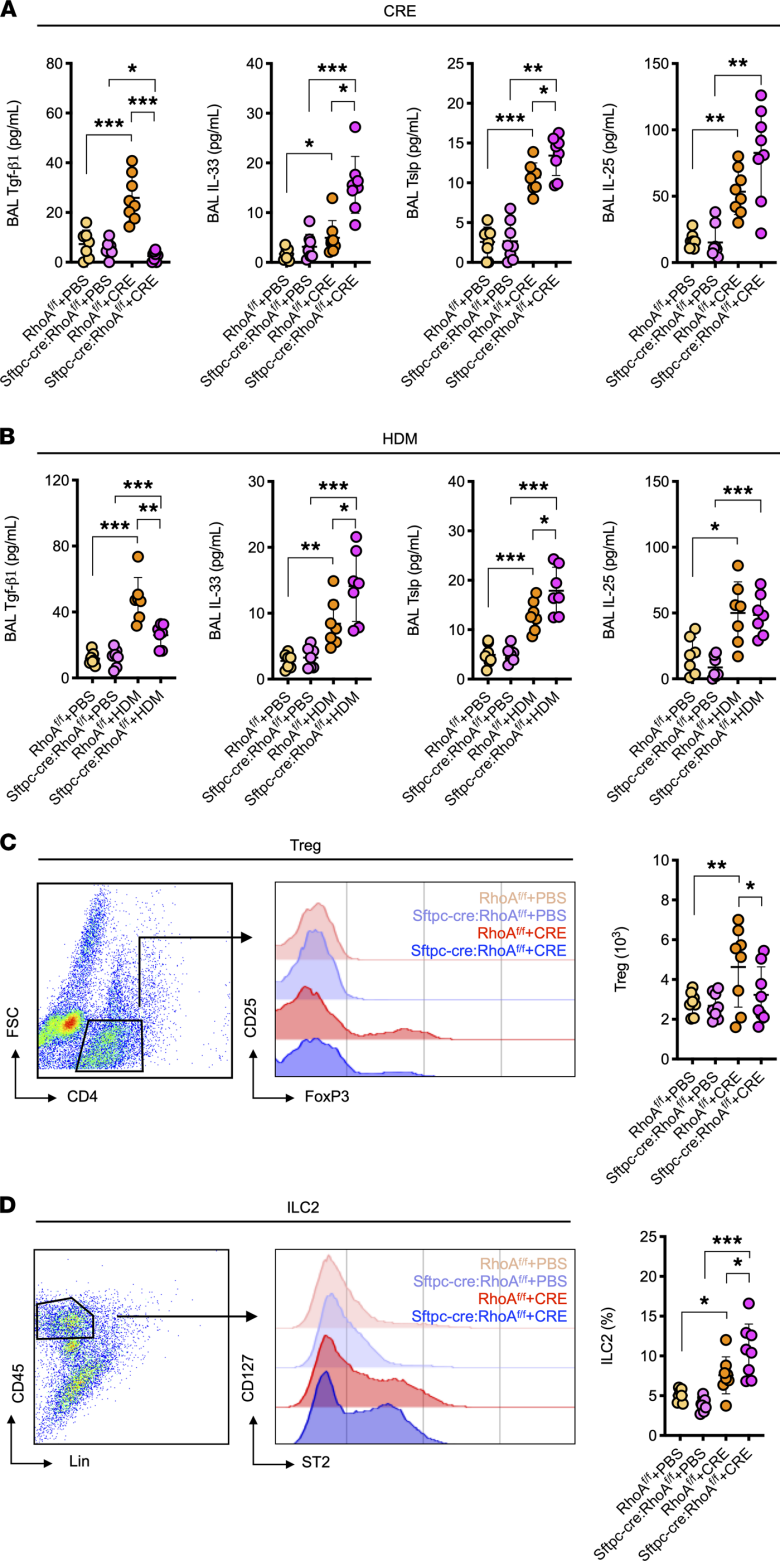
Deletion of RhoA leads to changes in cytokine release of asthma mouse models. (**A**) BALF levels of epithelial cell–derived cytokines in CRE-treated mice (*n* = 8). (**B**) BALF levels of epithelial cell–derived cytokines in house dust mite–treated (HDM-treated) mice (*n* = 7). (**C**) Number of Tregs (CD4^+^CD25^+^Foxp3^+^) in the lung tissues assessed by flow cytometry (*n* = 8). (**D**) Number of ILC2s (CD45^+^Lin^–^ST2^+^CD127^+^) in the lung tissues assessed by flow cytometry (*n* = 8). Data represent mean ± SEM of 2 independent experiments. Group comparisons were made using 2-way ANOVA. *P < 0.05, **P < 0.01, and ***P < 0.001.

Airway epithelial cells as the first line of defense against environmental allergens have an important role in determining the downstream immune responses by the release of inflammatory mediators (8, 28). To determine whether RhoA controls cytokine release in airway epithelial cells, we detected several major epithelial cell–derived cytokines in the BALF of asthma mouse models. Compared with PBS-treated mice, CRE-treated mice showed increased levels of Tgf-β1, Tslp, IL-25, and IL-33 in BALF. Intriguingly, the increased Tgf-β1 was nearly undetectable, but Tslp and IL-33 were further enhanced in BALF of CRE-treated *Sftpc-cre;RhoA^fl/fl^* mice (Figure 2A). These findings were further confirmed in a HDM-induced asthma model (Figure 2B). No significant difference was noted for IL-25 in those allergen-challenged mouse models between *RhoA^fl/fl^* and *Sftpc-cre;RhoA^fl/fl^* mice. Next, we examined whether the reduced Tgf-β1 subsequently affects downstream Tregs (CD4^+^CD25^+^FoxP3^+^) in the lung tissues of *Sftpc-cre;RhoA^fl/fl^* mice as determined by flow cytometry. Results revealed that CRE-challenged mice showed increased Tregs in the lung tissues, but *Sftpc-cre;RhoA^fl/fl^* mice exhibited decreased Tregs when compared with *RhoA^fl/fl^* mice (Figure 2C). In contrast to the reduced Tregs, we found that the CRE-challenged mice showed increased ILC2s (CD45^+^Lin^–^ ST2^+^CD127^+^) in the lung tissues, which was further enhanced in *Sftpc-cre;RhoA^fl/fl^* mice as compared with *RhoA^fl/fl^* mice (Figure 2D). No clear change was found for Th1, Th2, and Th17 cells in *Sftpc-cre;RhoA^fl/fl^* mice as compared with *RhoA^fl/fl^* mice (Supplemental Figure 5). Taken together, these findings indicate that RhoA in AT2 plays a distinct role in modulating Tgf-β1, Tslp, and IL-33 release that may contribute to the immune imbalance between Tregs and ILC2 cells.

**Figure 3:**
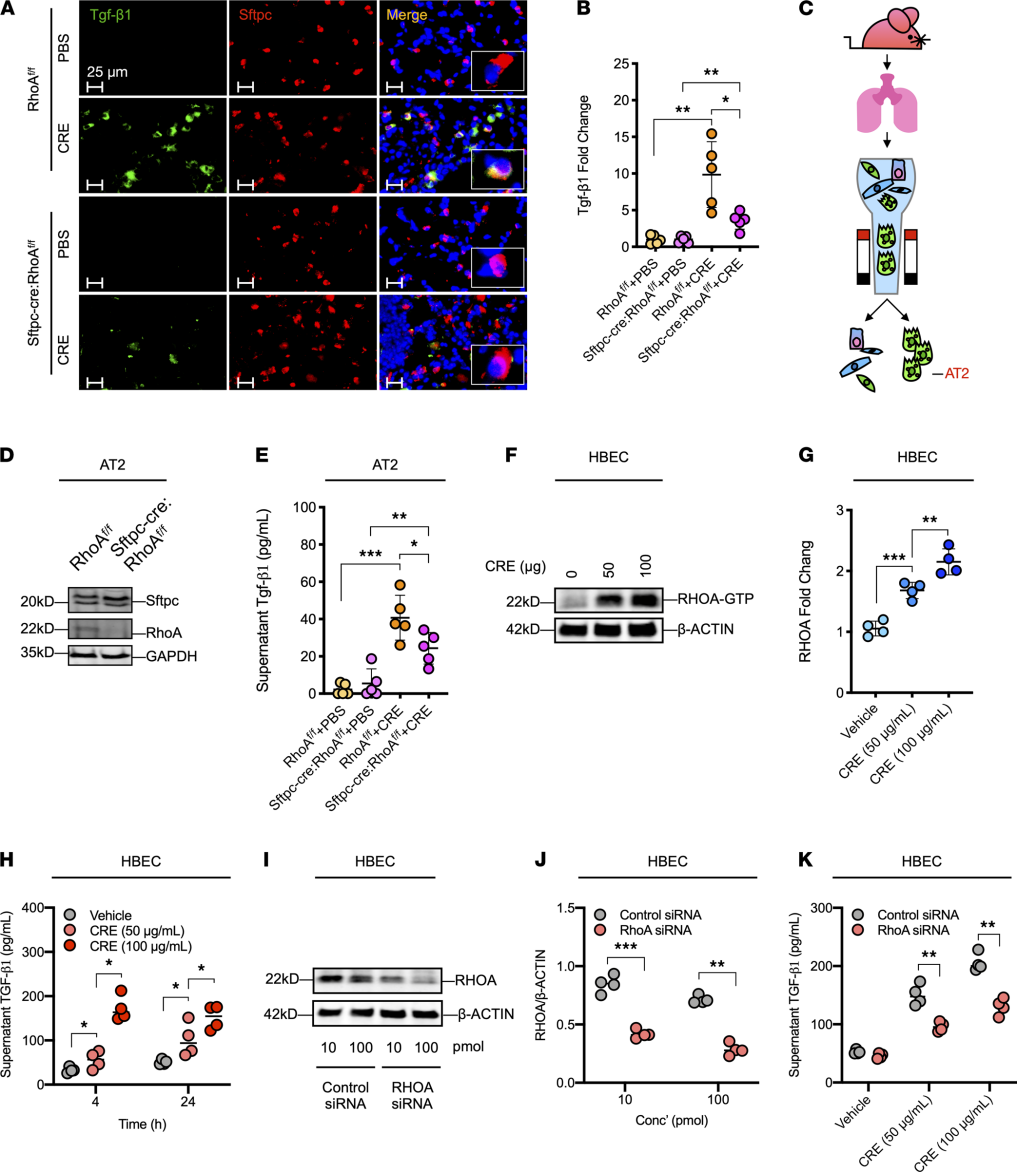
Deletion of RhoA leads to decreased Tgf-β1 release from AT2 cells. (**A**) Representative images of Tgf-β1 expression in AT2 cells of asthma mouse model by coimmunofluorescence staining with both Sftpc (red) and Tgf-β1 (green). (**B**) Quantification of Tgf-β1 expression in AT2 cells and expressed as fold changes in relative to PBS control (*n* = 5). (**C**) Schematic representation of AT2 cell isolation. (**D**) Confirmation of RhoA deletion in isolated AT2 cells of *Sftpc-cre;RhoA^fl/fl^* mice by Western blot. (**E**) Levels of Tgf-β1 in supernatants of CRE-treated AT2 cells isolated from WT and *Sftpc-cre;RhoA^fl/fl^* mice (*n* = 5). (**F**) Western bolt analysis of CRE-induced RHOA-GTP expression in HBECs. (**G**) Quantification of relative RHOA-GTP expression in **F** (*n* = 4). (**H**) Level of TGF-β1 in supernatants of CRE-treated HBECs (*n* = 4). (**I**) Confirmation of RHOA siRNA knockdown in HBECs by Western blot. (**J**) Quantification of relative RHOA expression in **I** (*n* = 4). (**K**) Level of TGF-β1 in supernatants of CRE-treated HBECs with control or RhoA siRNA knockdown (*n* = 4). Data represent mean ± SEM. Group comparisons were made using 2-way ANOVA (**B**, **E**, and **G**) and 2-tailed Student’s *t* test (**H** and **J**). *P < 0.05, **P < 0.01, and ***P < 0.001. Scale bars: 25 μm.

*AT2 cell–specific deletion of RhoA leads to decreased TGF-β1 release*. To confirm whether the reduced Tgf-β1 was caused by the deletion of RhoA in AT2 cells of *Sftpc-cre;RhoA^fl/fl^* mice, we examined Tgf-β1 expression specifically in AT2 cells of the asthma mouse model by coimmunofluorescence staining with both Tgf-β1 and Sftpc. TGF-β1 expression was sharply increased in AT2 cells of CRE-treated *RhoA^fl/fl^* mice but dramatically reduced in CRE-challenged *Sftpc-cre;RhoA^fl/fl^* mice as compared with *RhoA^fl/fl^* mice (Figure 3, A and B). To further confirm whether RhoA regulates Tgf-β1 release from AT2 cells, we performed in vitro analysis to measure Tgf-β1 in CRE-treated AT2 cells isolated from *RhoA^fl/fl^* and *Sftpc-cre;RhoA^fl/fl^* as illustrated in Figure 3C. The RhoA deficiency in isolated AT2 was confirmed by Western blot (Figure 3D). As expected, AT2 cells from *Sftpc-cre;RhoA^fl/fl^* mice secreted much lower levels of Tgf-β1 compared with those from *RhoA^fl/fl^* mice (Figure 3E). To determine whether the RhoA-regulated Tgf-β1 release from AT2 cells could be applied to airway epithelial cells, we used the human bronchial epithelial cells (HBECs) to validate the role of RHOA in regulating TGF-β1 release. In vitro studies revealed a dose-dependent CRE-induced activation of RHOA as assessed by Western blot for RHOA-GTP (Figure 3, F and G). Consistent with the RhoA activation, CRE induced TGF-β1 secretion from HBECs in a dose-dependent manner at 4 and 24 hour (Figure 3H). To see whether RHOA is directly associated with TGF-β1 release from HBECs, RHOA was knocked down in HBECs by using different doses of siRNA (10, 100 pmol) and then confirmed by Western blot (Figure 3, I and J). Notably, RHOA knockdown led to a significant reduction in the secretion of TGF-β1 from HBECs (Figure 3K), indicating that RHOA is critical in modulating TGF-β1 release from airway epithelium.

### rTGF-β1 reverses the increased airway inflammation in Sftpc-cre;RhoA^fl/fl^ mice.

**Figure 4:**
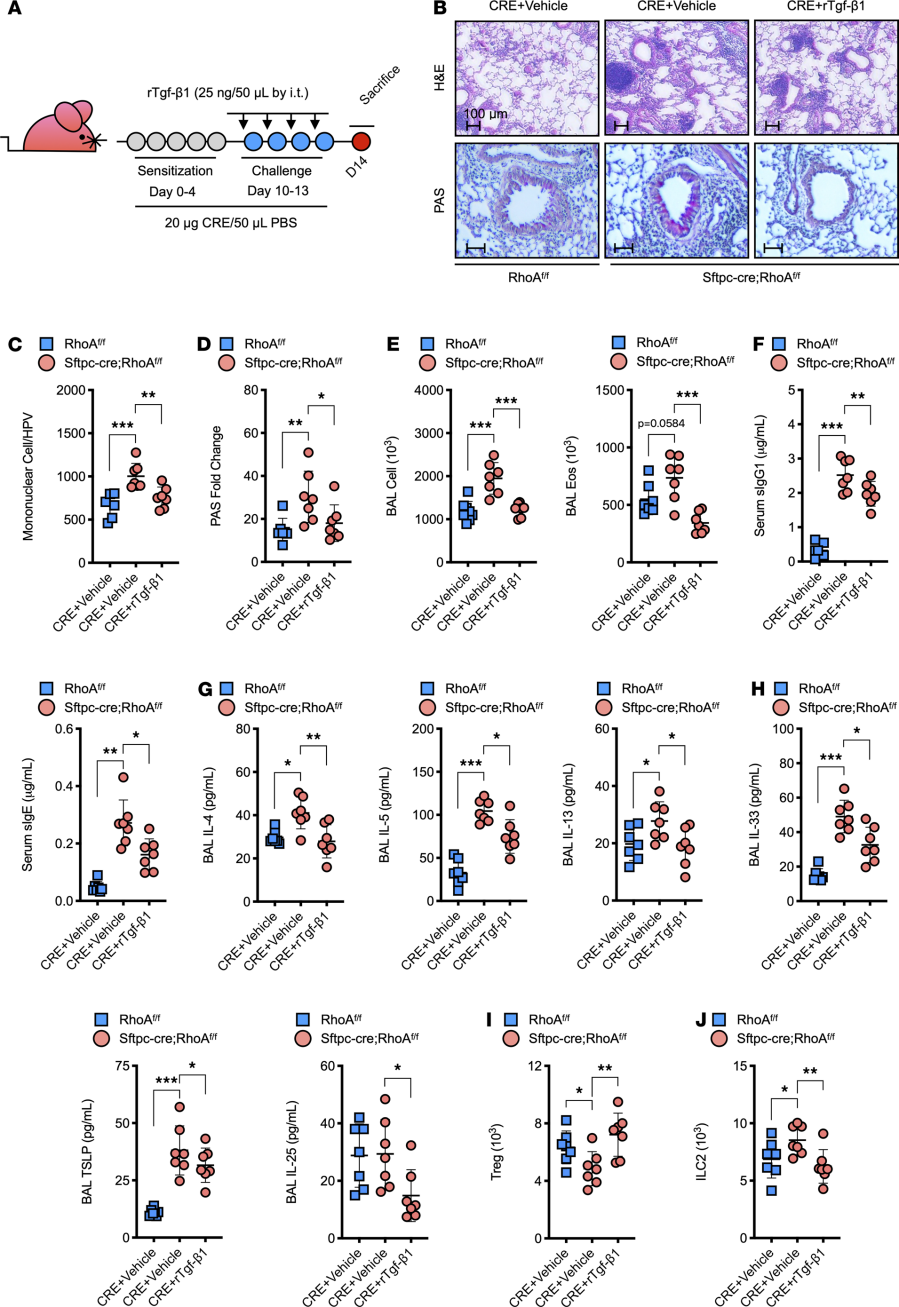
Recombinant Tgf-β1 reverses the increased allergic airway inflammation in *Sftpc-cre;RhoA^fl/fl^* mice. (**A**) Protocol for the treatment of CRE-challenged *Sftpc-cre;RhoA^fl/fl^* mice with recombinant Tgf-β1(rTgf-β1) by i.t. administration. (**B**) Histological examination of mouse paraffin lung sections stained with H&E (upper panel) and PAS (lower panel). (**C**) Quantification of mononuclear cell infiltrates in H&E-stained lung sections (*n* = 7). (**D**) Goblet cells quantification in PAS-stained lung sections (*n* = 7). (**E**) BALF total and eosinophil cell counts as determined by flow cytometry (*n* = 7). (**F**) Serum levels of CRE-specific IgE and IgG1 (*n* = 7). (**G** and **H**) BALF levels of Th2 cytokines (**G**) and epithelial cell–derived cytokines (**H**) (*n* = 7). (**I** and **J**) Percentage of Treg (CD4^+^CD25^+^Foxp3^+^) (**I**) and ILC2s (CD45^+^Lin^–^Thy1.2^+^GATA3^+^) (**J**) in the lung tissues assessed by flow cytometry (*n* = 7). Data represent mean ± SEM of 2 independent experiments. Group comparisons were made using 2-way ANOVA (**C**–**J**). *P < 0.05, **P < 0.01, and ***P < 0.001. Scale bars: 100 μm.

Given that RhoA deletion in AT2 almost abolished the secretion of Tgf-β1, we hypothesized that the increased lung inflammation we observed in *Sftpc-cre;RhoA^fl/fl^* mice might be due to the lack of Tgf-β1. To test the possibility, Tgf-β1 recombinant protein (25 ng/mouse) was administrated via intratracheal on day 9 before allergen challenge following the protocol illustrated in Figure 4A. As expected, administration of rTgf-β1 significantly reversed the increased inflammatory cell recruitment into the airways and peribronchial (Figure 4, B and C; H&E) and goblet cell hyperplasia (Figure 4, B and D; PAS) observed in *Sftpc-cre;RhoA^fl/fl^* mice. Total inflammatory cells — particularly eosinophils (Figure 4E) and not neutrophils (Supplemental Figure 3C) in the BALF, and CRE-specific IgE and IgG1 in serum of *Sftpc-cre;RhoA^fl/fl^* mice (Figure 4F) — were significantly reduced following treatment with TGF-β1. IL-4, IL-5, and IL13 in BALF were also significantly reduced in these TGF-β1–treated *Sftpc-cre;RhoA^fl/fl^* mice (Figure 4G). Furthermore, reduced levels of IL-33, Tslp, and IL-25 were detected in BALF of rTgf-β1–treated mice (Figure 4H). Consistently, we found that rTgf-β1 administration reversed the decreased CD4^+^CD25^+^FoxP3^+^ Tregs (Figure 4I) and increased ILC2s in the lung tissues of *Sftpc-cre;RhoA^fl/fl^* mice (Figure 4J). The results indicate that treatment with Tgf-β1 reversed the increased allergic airway inflammation in *Sftpc-cre;RhoA^fl/fl^* mice.

### RhoA-dependent transcriptional programs in AT2 cells.

**Figure 5:**
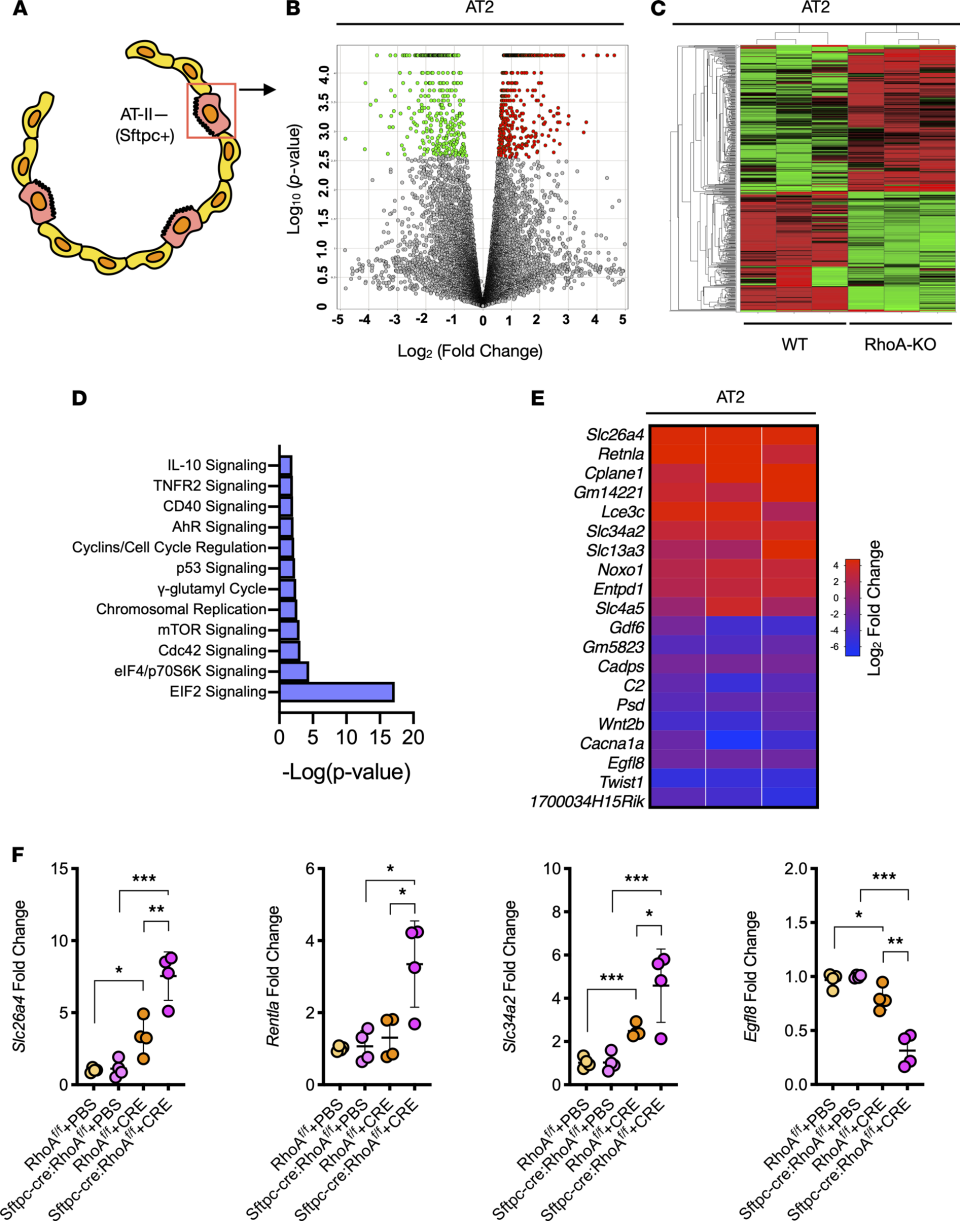
RhoA-dependent transcriptional programs in AT2 cells. (**A**) Schematic representation of AT2 cells used for RNA-seq analysis. (**B**) Volcano plot of differentially expressed genes in AT2 cells isolated from WT versus *Sftpc-cre;RhoA^fl/fl^* mice that were treated with CRE. The logarithms of the fold changes of individual genes (*x* axis) are plotted against the negative logarithm of their *P* value to base 10 (*y* axis). (**C**) Heatmap of differentially expressed mRNAs in AT2 cells (FDR-corrected *P* < 0.05). (**D**) Top enriched categories of canonical pathways identified by IPA. (**E**) Top 10 up- or downregulated genes in AT2 cells. (**F**) qPCR analysis of differentially expressed genes of interest in AT2 cells. Gene expression was normalized to actin and expressed as fold change over untreated control samples (*n* = 4). Data represent mean ± SEM. Group comparisons were made using 2-way ANOVA (**F**). *P < 0.05, **P < 0.01, and ***P < 0.001.

To further investigate the mechanisms by which RhoA regulated Tgf-β1 release and allergic airway inflammation, we analyzed the transcriptional profiles of AT2 cells (Figure 5A) isolated from the lungs of *RhoA^fl/fl^* and *Sftpc-cre;RhoA^fl/fl^* mouse models of asthma as illustrated in Figure 3C. Results revealed approximately 450 mRNAs that were differentially expressed in AT2 cells between *RhoA^fl/fl^* and *Sftpc-cre;RhoA^fl/fl^* mice (FDR-corrected *P* < 0.05), including 203 downregulated and 247 upregulated genes. The pattern of the up- and downregulated genes in AT2 cells is illustrated by a volcano plot (Figure 5B) and heatmap (Figure 5C). The differentially expressed genes were then categorized into related canonical pathways. Several top canonical pathways were identified, including EIF2, eIF/p70S6K, Cdc42, mTOR, and AhR signaling pathways (Figure 5D). Further results revealed the most upregulated (e.g., *Slc26a4*, *Retnal*, *Lce3c*, *Slc34a2*, and *Noxo1*) and downregulated genes (e.g., *Gpnmb* and *Twist1*) (Figure 5E). The most up- or downregulated genes were selected for further validation by quantitative PCR (qPCR). The results confirmed increased expression of *Slc26a4*, *Retnal*, and *Slc34a*, and decreased *Egfl8* in AT2 cells from *Sftpc-cre;RhoA^fl/fl^* mice as compared with those from WT mice (Figure 5F). Of those, the solute carrier family 26, member 4 (Slc26a4), a Cl-conductance–related gene transcript (29), was the most upregulated gene in RhoA-deficient AT2 cells.

### AhR regulates Slc26a4 expression.

**Figure 6:**
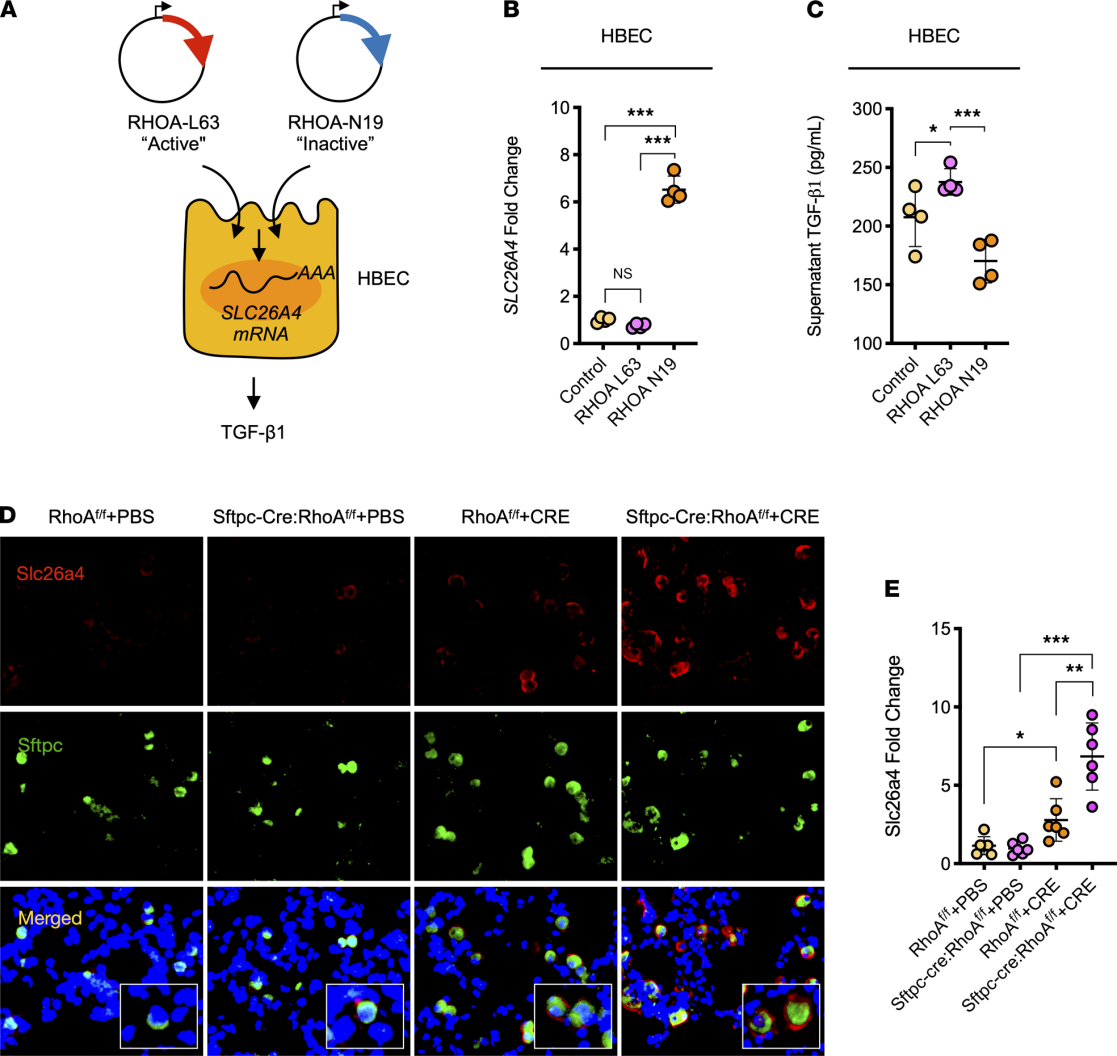
RhoA regulates SLC26A4 expression. (**A**) Schematic representation of HBECs expressing either a constitutively active RHOA (RHOA-L63) or dominant-negative (RHOA-N19) for SLC26A4 regulation by RhoA and subsequently TGF-β1 secretion. (**B**) qPCR analysis of relative *SLC26A4* expression in HBECs expressing either RHOA-L63 or RHOA-N19 (*n* = 4). (**C**) Level of TGF-β1 in supernatants of RHOA-L63– or RHOA-N19–transfected HBECs treated with CRE (*n* = 4). (**D**) Representative immunofluorescence images of Slc26a4 (red) expression in lung tissue AT2 cells (Sftpc, green) of CRE-induced mouse model of asthma with *RhoA^fl/fl^* or *Sftpc-cre;RhoA^fl/fl^* mice. (**E**) Quantification of relative Slc26a4 expression in **E** (*n* = 6). Data represent mean ± SEM. Group comparisons were made using 2-way ANOVA (**B**, **C**, and **E**). *P < 0.05, **P < 0.01, and ***P < 0.001. Total original magnification, ×20.

To further confirm the regulation of SLC26A4 expression by RHOA, we transfected HBECs with either a constitutively active RHOA (RHOA-L63) or dominant-negative (RHOA-N19) (Figure 6A). As expected, HBECs expressing RHOA-N19 showed increased expression, but HBECs expressing RHOA-L63 exhibited decreased expression, of SLC26A4 when compared with controls in response to CRE (Figure 6B). Consistently, TGF-β1 in supernatants was significantly reduced in HBECs expressing RHOA-N19 but increased in cells expressing RHOA-L63 (Figure 6C). Furthermore, the increased expression of SLC26A4 was also found in AT2 cells of our asthma mouse model as assessed by coimmunostaining of Slc26a4 and Sfptc (Figure 6, D and E). Notably, the increased Slc26a4 was further enhanced in AT2 cells of CRE-treated *Sftpc-cre;RhoA^fl/fl^* mice compared with control mice. Together, these data suggest a possible mechanism that RHOA regulates TGF-β1 secretion from airway epithelial cells through modulating SLC26A4 expression.

### Increased SLC26A4 in AT2 cells and serum of asthmatic patients.

**Figure 7:**
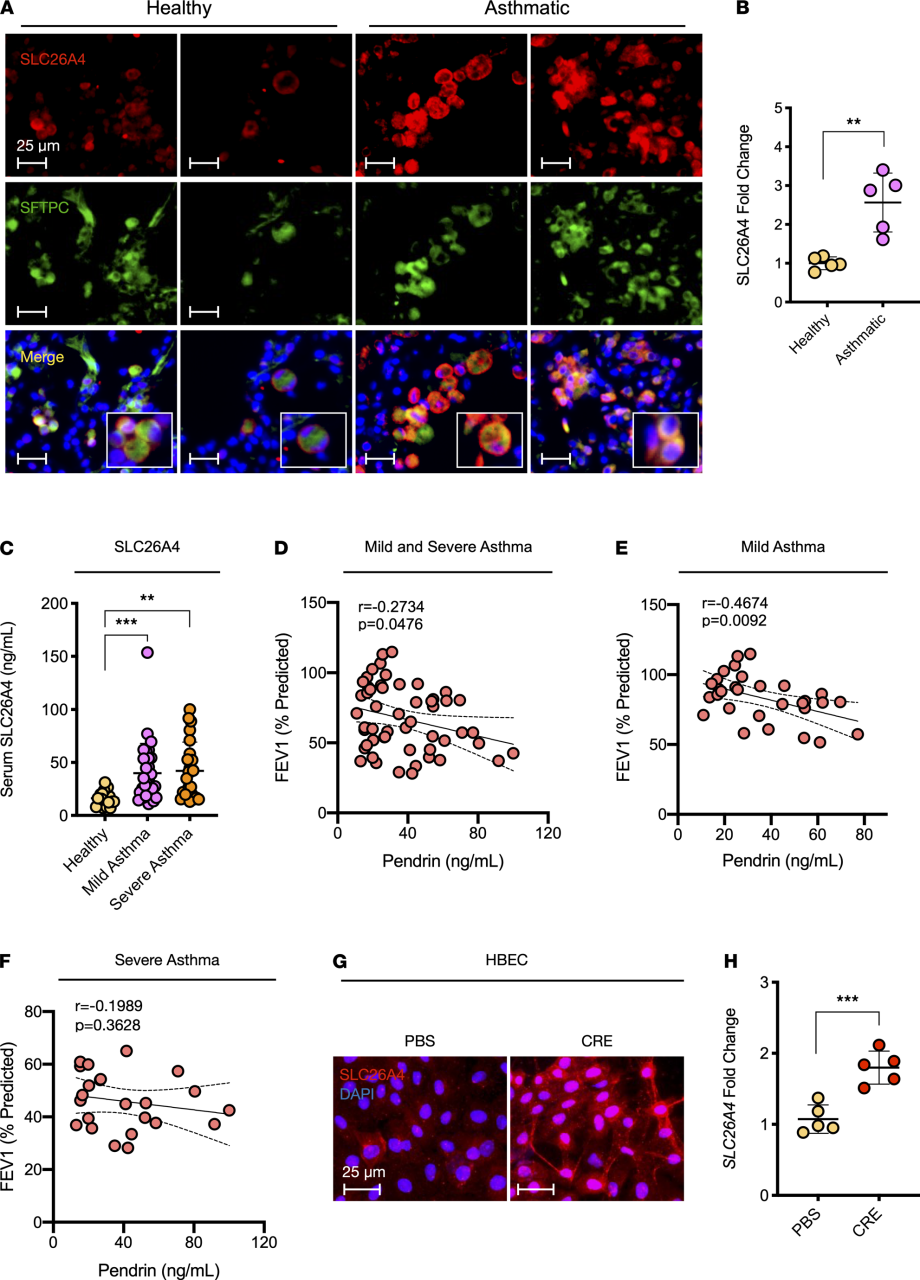
Increased SLC26A4 in AT2 cells and serum of asthmatic patients. (**A**) Representative immunofluorescence images of SLC26A4 (red) expression in lung tissue AT2 cells (SFTPC, green) of asthmatics and healthy controls. (**B**) Quantitative analysis of SLC26A4 expression in **A** (*n* = 5). (**C**) Serum levels of SLC26A4 in healthy subjects (*n* = 18) and patients with mild (*n* = 31) and severe (*n* = 24) asthma. (**D**) Correlation between serum levels of SLC26A4 (pendrin) and FEV_1_% among asthmatics (*n* = 55). (**E** and **F**) Correlation between serum levels of SLC26A4 (pendrin) and FEV_1_% among the mild (**E**, *n* = 31) and severe (**F**, *n* = 23) asthmatics, respectively. (**G**) Representative images of SLC26A4 expression in HBECs after CRE treatment. (**H**) Quantification of SLC26A4^+^ staining in **G** (*n* = 5). Data represent mean ± SEM. Group comparisons were made using 2-tailed Student’s *t* test (**B** and **H**) and 2-way ANOVA (**C**). *P < 0.05, **P < 0.01, and ***P < 0.001. Scale bars: 25 μm.

SCL26A4 has been shown to be increased in endobronchial biopsies from patients with asthma (30–32), but whether SCL26A4 is increased or decreased in AT2 cells remains unclear. Consistent with the previous report, we found that SLC26A4 expression was increased in the lung tissues of patients with asthma. Particularly, there was a significant increase in the expression of SLC26A4 in AT2 cells of asthmatic patients compared with healthy controls, as assessed by coimmunostaining of SLC26A4 and SFTPC (Figure 7, A and B). Furthermore, higher levels of SLC26A4 were detected in serum from mild and severe asthmatics when compared with healthy controls, but no difference was detected between mild and severe asthmatics (Figure 7C). Next, we investigated the correlation between serum levels of SLC26A4 and pulmonary function parameters (FEV_1_%, FEF_25–75_%, FVC%, and FEV_1_/FVC) among asthmatic patients (Supplemental Table 2). Particularly, a significantly negative correlation was found for serum levels of SLC26A4 and FEV_1_% among asthmatics (*r* = –0.2734, *P =* 0.0476, *n* = 53, Figure 7D), and the significance was further strengthened when the analysis was limited to the mild-moderate patients (*r* = –0.4674, *P* = 0.0092, *n* = 30, Figure 7E). However, there was no statistical significance between serum levels of SLC26A4 and FEV_1_% among the severe patients (*r* = –0.1989, *P* = 0.3628, *n* = 23, Figure 7F). Lastly, we examined whether SLC26A4 in human airway epithelial cells can be activated by CRE. As expected, we found an increased expression of SLC26A4 in HBECs after treatment with CRE for 24 hours (Figure 7, G and H). Collectively, these results suggest an increased SLC26a4 in AT2 cells and serum of asthmatic patients, which may be critical in asthma.

### Inhibition of airway chloride channel diminishes allergic airway information.

**Figure 8:**
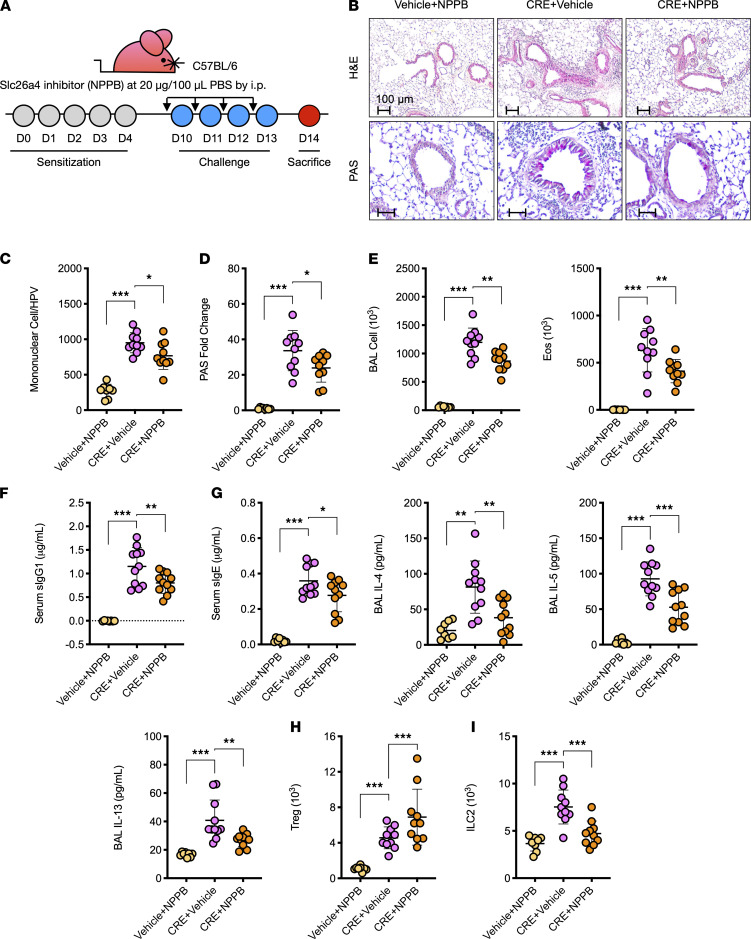
Inhibition of airway chloride channel diminishes allergic airway information. (**A**) Protocol for the treatment of CRE-induced asthma mouse model with NPPB. (**B**) Histological examination of mouse paraffin lung sections stained with H&E (upper panel) and PAS (lower panel). (**C**) Quantification of mononuclear cell infiltrates in H&E-stained lung sections (*n* = 8–10). (**D**) Goblet cells quantification in PAS-stained lung sections (*n* = 8–10). (**E**) BALF total and eosinophil cell counts as determined by flow cytometry (*n* = 8–10). (**F**) Serum levels of CRE-specific IgE and IgG1 (*n* = 8–10). (**G**) BALF levels of Th2 cytokines (*n* = 8–10). (**H** and **I**) Numbers of Treg (CD4^+^CD25^+^Foxp3^+^) (**H**) and ILC2s (CD45^+^Lin^–^Thy1.2^+^GATA3^+^) (**I**) in the lung tissues assessed by flow cytometry (*n* = 8–10). Data represent mean ± SEM of 2 independent experiments. Group comparisons were made using 2-way ANOVA (**C**–**I**). *P < 0.05, **P < 0.01, and ***P < 0.001. Scale bars: 100 μm.

Next, we investigated whether Slc26a4 plays a role in modulating airway allergic inflammation by using the chloride channel inhibitor 5-Nitro-2-(3-phenylpropylamino) benzoic acid (NPPB), which has been shown to inhibit Slc26a4-induced Cl^–^ uptake (33) and Slc26a4 activity (34). NPPB was administrated via i.p. injection 30 minutes before allergen challenge following the protocol illustrated in Figure 8A. Pretreatment of mice with NPPB reduces CRE-induced airway inflammation based on H&E and periodic acid–Schiff (PAS) staining of lung tissue sections, which showed a reduction of inflammatory cell recruitment into the airways and peribronchial (Figure 8, B and C; H&E) and goblet cell hyperplasia (Figure 8, B and D; PAS). Total inflammatory cells, mainly eosinophils (Figure 8E) but not neutrophils (Supplemental Figure 3D), in the BALF. Furthermore, CRE-specific IgE and IgG1 in serum of *Sftpc-cre;RhoA^fl/fl^* mice were significantly inhibited following NPPB treatment (Figure 8F). NPPB treatment also inhibited the CRE-induced increased Th2 cytokines IL-4, IL-5, and IL-13 in BALF (Figure 8G). When Tregs and ILC2s in the lung tissues were examined, NPPB further promoted the increased Tregs (Figure 8H) but inhibited the increased ILC2s (Figure 8I) in the lung tissues of CRE-treated mice. The results indicate that Slc26a4 as an airway Cl^–^ channel gene may play an important role in allergic airway inflammation.

### SLC26A4 regulates epithelial cytokine release.

**Figure 9:**
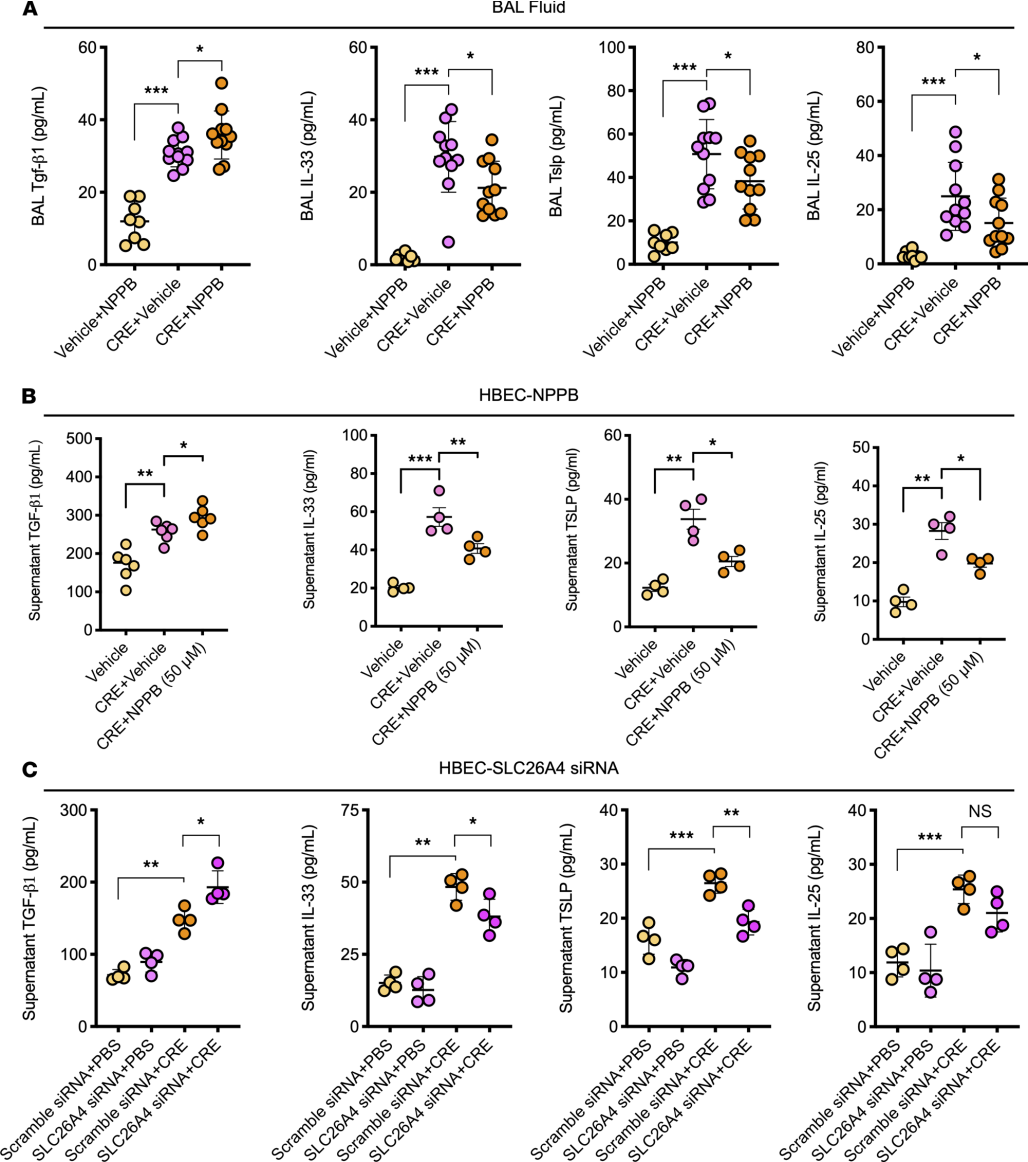
SLC26A4 regulates epithelial cytokine release. (**A**) Epithelial cell–derived cytokines in the BALF of NPPB treated mice (*n* = 8–10). (**B**) Epithelial cell–derived cytokines in supernatants of CRE-treated HBECs in the presence or absence of NPPB treatment (*n* = 4–6). (**C**) Epithelial cell–derived cytokines in supernatants of CRE-treated HBECs with or without SLC26A4 knockdown (*n* = 4). Data represent mean ± SEM of 2 independent experiments. Group comparisons were made using 2-way ANOVA (**A**–**C**). *P <0.05, **P < 0.01, and ***P < 0.001.

Given the significance of Slc26a4 in airway inflammation, our further study investigated whether Slc26a4 plays a role in modulating epithelial cytokine release. We detected epithelial cell–derived cytokines in BALF of NPPB-treated mice. We found that pretreatment of mice with NPPB further enhanced the increased Tgf-β1 but inhibited the increased IL-33, Tslp, and IL-25 in BALF (Figure 9A). To confirm the role of SLC26A4 in regulating cytokine release from epithelial cells, we performed in vitro analysis in HBECs by using SLC26A4 inhibitor NPPB. CRE-treated HBECs showed increased levels of TGF-β1, IL-33, TSLP, and IL-25 (Figure 9B); pretreatment with NPPB further promoted CRE-induced TGF-β1 but inhibited the increased IL-33, TSLP, and IL-25 secretion. These findings were further supported by SLC26A4 knockdown in HBECs. SLC26A4 knockdown in HBECs was confirmed by Western blot and presented in Supplemental Figure 6. SLC26A4 knockdown showed increased TGF-β1 but decreased IL-33, TSLP, and IL-25 as compared with WT HBECs after exposed to CRE (Figure 9C). The results indicate that SLC26A4 regulates epithelial cell–derived cytokine release, perhaps through independent regulatory mechanisms.

## Discussion

Here, we report that RhoA in AT2 protects against allergen-induced airway inflammation in asthma and its underlying mechanisms. In particular, using *Sftpc-cre;RhoA^fl/fl^* mice, we found that AT2 cell–specific deletion of RhoA leads to exacerbation of allergen-induced AHR and airway inflammation with elevated Th2 cytokines and eosinophils in BALF. Most importantly, we investigated its underlying mechanisms by which RhoA in AT2 regulates allergic airway inflammation. We demonstrated that disruption of RhoA in AT2 blocked the secretion of Tgf-β1, which limited the development of Tregs in the lung tissues and subsequently led to the increased allergic airway inflammation. This was indeed supported by the fact that administration of Tgf-β1 reversed the increased allergic airway inflammation observed in *Sftpc-cre;RhoA^fl/fl^*. Furthermore, we identified differentially expressed genes in AT2 cells that may play an important role in RhoA-mediated allergic inflammation by performing RNA-seq analysis. Among these genes, Slc26a4, a transcript involved in Cl^–^ conductance, is one of the most upregulated genes in RhoA-deficient AT2 cells. Of interest, SLC26A4 is increased in AT2 cells and serum of asthmatic patients and is associated with lung function, and inhibition of SLC26A4 promoted epithelial cell TGF-β1 release and attenuated allergic airway information in asthma.

In this study, we used *Sftpc-cre;RhoA^fl/fl^* mice to see the role of RhoA specifically in AT2 in allergic airway inflammation. However, this study was limited by the lack of genetic markers for AT2 cells. We used Sftpc because Sftpc is expressed at high levels by AT2 cells in the lung and has been widely used as a marker for AT2 cells (35–37). However, studies have also shown that Sftpc is not only expressed in AT2 cells, but is also present in embryonic lung epithelial progenitors and mature airway epithelial cells (38–40). We detected Sftpc expression in lung epithelial cells of our newly generated *Sftpc-cre;RhoA^fl/fl^* mice. We found that Sfptc is expressed at the highest levels by AT2 cells but is also seen in basal cells. Thus, although we focused our studies on AT2 cells, the mechanisms about the RhoA-involved regulation of AT2 cells may also apply to other epithelial cells. Importantly, future investigations are essential with single-cell RNA-seq analysis and lineage-tracing experiments to identify different AT2 subtypes and their unique markers to further define the role of AT2 cells in allergic airway inflammation.

RhoA is an intracellular signal transducer of the Rho family of small GTPases (41). Increased activation of RhoA/Rho-kinase has been associated with bronchial smooth muscle contraction and AHR in asthma (16, 17). Furthermore, the existing data have suggested a significant role for RhoA in regulating airway inflammation through affecting migration, differentiation, and function of various inflammatory cells (e.g., eosinophils, macrophages, mast cells, and T cells; refs. 14, 22, 42–49). RhoA/Rho-kinase also plays a critical role in mast cell morphology and degranulation (50). Earlier works have demonstrated that disruption of RhoA in T cells inhibited Th2/Th17 differentiation and activation and protected against allergic airway inflammation (14, 22), suggesting that downregulation of RhoA/ROCK signaling prevents allergic airway inflammation. Thus, RhoA has been considered as a novel target molecule for the treatment of asthma (11, 18). On the contrary, our present work shows that deletion of RhoA specifically in AT2 cells causes an increase in allergen-induced Th2-associated airway inflammation. These conflicting findings suggest a datagram of RhoA duality in its role to both promote and inhibit allergic airway inflammation, depending on a variety of factors, including cell types, RhoA downstream targets, and balancing of innate or adaptive immunity. Thus, additional detailed investigations of RhoA’s function in different cell types at varying stages of the disease development and progression are clearly needed to properly assess the potential utility of RhoA as a bona fide target for modulation.

Our work reveals that active RhoA signaling in AT2 cells controls cell activation and inflammatory cytokine release that may affect the downstream balancing of innate or adaptive immunity. We observed a marked increase in the levels of epithelial cell–derived cytokines Tgf-β1, Tslp, IL-25, and IL-33 in BALF of allergen-induced mouse models of asthma. However, mice with RhoA deletion in AT2 cells showed almost complete abolishment of Tgf-β1 secretion but promoted Tslp, IL-25, and IL-33 production. These findings suggest an immunoimbalance between decreased Tgf-β1 and augmented Tslp, IL-25, and IL-33 secretion, supporting that lack of Tgf-β1 may contribute to the increased airway inflammation in *Sftpc-cre;RhoA^fl/fl^* mice. Indeed, our findings imply a feed-forward connection between Tgf-β1 and RhoA signaling in AT2 cells. We found reduced Tgf-β1 in supernatants of AT2 cells isolated from CRE-treated *Sftpc-cre;RhoA^fl/fl^* mice by in vitro analysis and reduced expression of Tgf-β1 in the lung tissue AT2 cells of CRE-treated *Sftpc-cre;RhoA^fl/fl^* mice. Some of studies found that Sftpc is also expressed in other epithelial cells (38–40), raising the possibility that the role of RhoA in protecting against allergic inflammation may not limit only to AT2 cells. This, indeed, was supported by our in vitro study by using HBECs. CRE-induced expression of RhoA-GTP and secretion of TGF-β1 in human primary airway epithelial cells, as well as RhoA knockdown, led to a significant reduction of TGF-β1 secretion from airway epithelium. Taken together, our studies suggest that RhoA controls the release of Tgf-β1 from AT2 cells that may contribute to the development of allergic airway inflammation.

TGF-β1 is a multifunctional cytokine that plays an important role in cell growth, differentiation, and immune regulation (51–55). However, the exact role of epithelial cell–derived TGF-β1 in allergic asthma is still unknown. It was evident that TGF-β1 induces expression of FOXP3 and converts conventional CD4^+^CD25^–^ T cells to induced FOXP3^+^ Tregs that mediate immune suppression in vivo (52, 56). Thus, Tregs may be one of the key mechanisms in mediating Tgf-β1–induced immune suppression. Indeed, we found a significant reduction of Tregs in the lung tissues of allergen-challenged *Sftpc-cre;RhoA^fl/fl^* mice, and pretreatment with Tgf-β1 recombinant protein reversed the increased inflammation observed in *Sftpc-cre;RhoA^fl/fl^* mice with increased lung tissue CD4^+^CD25^+^FoxP3^+^ Tregs. These findings support the rationale that Tgf-β1 promotes naive T cell differentiation into Tregs and subsequently inhibits allergic airway inflammation.

Another possible mechanism elucidated from this study is that Tgf-β1 may inhibit ILC2s either directly or through TGF-β1–induced Tregs. ILC2s are important sources of Th2 cytokines such as IL-5 and IL-13 that contribute to the increased Th2-associated airway inflammation observed in asthma (57). Increased ILC2 cells have been considered as one of the major phenotypes associated with Th2-associated airway inflammation (58). It was reported that human ILC2s express a TGF-β type II receptor (TGFBR2), and TGF-β1 strongly suppresses the activation of human ILC2s (59). Tregs have been considered to be potent regulators of ILC2s due to their role in suppressing ILC2 function (60, 61). Although there is no direct evidence, our studies suggest a possible inverse relationship between Tgf-β1 or lung tissue Tregs and ILC2 cells. However, epithelial cell–derived Tgf-β1 has also been shown to be a critical cofactor for the expansion and activation of ILC2s (54). Thus, Tslp, IL-33, and IL-25 secretion may be independent of the RhoA/Tgf-β1/Treg axis and modulate ILC2 phenotype and function (62), which would be of interest to study in the future.

To further uncover the underlying mechanisms by which RhoA regulated Tgf-β1 release and allergic airway inflammation, we analyzed the transcriptional profiles of WT versus RhoA-deficient AT2 cells and identified a total of 450 genes differentially regulated by RhoA in AT2 cells. Ingenuity Pathway Analysis (IPA; Qiagen) based on these differentially expressed genes showed enrichment for the eIF2 and eIF4/p70S6K signaling pathways, which regulate mRNA translation and subsequent protein synthesis during normal conditions and in response to environmental stress (63). Both eIF2α subunit and eIF4 have been shown to be associated with TGF-β1 receptors and regulate TGF-β1 signaling transduction (64, 65). In addition, Cdc42, mTOR, and AhR signaling were also the major canonical pathways regulated by RhoA. Further analysis identified the genes that are most upregulated (e.g., *Slc26a4*, *Retnal*, *Lce3c*, *Slc34a2*, and *Noxo1*) or downregulated (e.g., *Egfl8* and *Twist1*) in RhoA-deficient AT2 cells. Of these, *Slc26a4* gene, encoding an anion exchange protein called pendrin, is involved primarily in transmembrane transport of small molecules, including chloride, iodide, and bicarbonate (66). Increased expression of *SLC26A4* has been associated with allergic airway inflammation, hyperreactivity, and increased mucus production (29, 67, 68). The regulation of Slc26a4 expression in AT2 cells by RhoA was further validated by an in vivo mouse model with *Sftpc-cre;RhoA^fl/fl^* mice and by in vitro analyses in HBECs expressing constitutively active RHOA-L63 or dominant-negative RHOA-N19. Importantly, our human study demonstrated that SLC26A4 expression was increased in AT2 cells of asthmatic patients, and demonstrated for the first time to our knowledge that serum levels of SLC26A4 were much higher in patients with mild and severe asthma compared with healthy controls. Furthermore, a significantly negative correlation was observed for serum levels of SLC26A4 and FEV_1_ when analyses were performed for serum levels of SLC26A4 and pulmonary function parameters among asthmatic patients. These findings were consistent with a recent report that SLC26A4 was elevated in human BALF of patients with acute respiratory distress syndrome (ARDS) compared with the control subjects (69). However, the exact function of the soluble form of SLC26A4 in airway inflammation has been well studied for SLC26A4 as a membrane-associated anion transporter, but it remains unknown; this would be of interest to pursue in the future. Together, our study suggests that SLC26A4 may have the pathogenic significance in the development of asthma.

Indeed, a study has shown that inhibition of *SLC26A4* suppressed AHR and mucin expression (70). Thus, we used NPPB, an inhibitor of anion channels that has been shown to suppress Slc26a4-induced Cl^–^ uptake (33) and Slc26a4 activity (34), to explore whether Slc26a4 is a potential molecular target for asthma treatment. As expected, pretreatment of mice with NPPB abrogated CRE-induced airway inflammation and Th2-associated cytokines. To further explore the role of Slc26a4 in airway inflammation, we examined whether SLC26A4 is involved in regulating epithelial cytokine release. Indeed, pretreatment of mice with NPPB showed further increased Tgf-β1 but reduced IL-33, Tslp, and IL-25 levels in BALF. The same pattern was supported by our in vitro analyses in pretreated HBECs with NPPB or in HBEC with SLC26A4 knockdown. These results indicate that a specific blockade of SLC26A4 may open innovative therapeutic avenues in the treatment of asthma. However, the exact role of the anion transporter regarding the pathogenesis of asthma is still unclear, thus warranting further, in-depth investigations of SLC26A4 function in AT2 cells.

Together, our data demonstrate a unique role for RhoA in AT2 cells in regulating allergen-induced Th2-associated airway inflammation. RhoA KO in AT2 cells may limit Tgf-β1 release that contributes to the increased allergic airway inflammation. Mechanistically, we analyzed the transcriptional profiles by RNA-seq and identified Slc26a4 as one of the RhoA-regulated targets in AT2 cells. Further studies suggest that Slc26a4 as a transcript involved in Cl^–^ conductance plays an important role in epithelial cytokine release and allergic airway information through regulating the immune balance between Tregs and ILC2s. While there is no a direct evidence for the increased airway inflammation caused by the immunoimbalance between Tregs and ILC2s, a rich body of literature supports that Tregs is one of the key mechanisms in mediating Tgf-β1–induced immune suppression (52, 56). In contrast, epithelial cell–derived cytokines, specifically Tslp, IL-33, and IL-25, directly modulate ILC2 phenotype and function and subsequently promote allergic airway inflammation (57, 58). Importantly, TGF-β1/Treg has been shown to suppress the activation of human ILC2s (59–61). Thus, our study suggests a potentially novel mechanism that the RhoA/SLC26A4 axis in AT2 cells protects against allergic airway inflammation through the immunoimbalance between augmented Treg and reduced ILC2 cells. Collectively, these studies highlight a critical role for the activated RhoA signaling in AT2 cells in allergic airway inflammation and provide a potential therapeutic effect of anti–RhoA-SLC26A4 approaches in patients with asthma.

## Methods

### Mice.

*Sftpc-cre;RhoA^fl/fl^* mice on the C57BL/6 background were generated by cross-breeding *Sftpc-cre* with *RhoA^fl/fl^* mice. *Sftpc-cre* mice were provided by Michael A. O’Reilly at the University of Rochester Medical Center (Rochester, New York, USA), and *RhoA^fl/fl^* mice were provided by Yi Zheng at the University of Cincinnati (Cincinnati, Ohio, USA). All mice were maintained under specific pathogen–free conditions at the animal facility of the Johns Hopkins University School of Medicine.

### Allergen-induced asthma mouse model.

Asthma mouse model induced by cockroach (CRE, Greer Laboratories Inc.) and HDM exact (Stallergenes Greer) was established as previously reported (71). Mice were sacrificed, and samples were collected on the next day after the last allergen challenge. In a separate experiment, mice were treated with 25 ng recombinant mouse TGF-β1 (BioLegend) dissolved in PBS by intratracheal administration or with 20 μg 5-nitro-2-[(3-phenylpropyl)amino]benzoic acid (NPPB, CAS# 107254-86-4) by i.p. injection 30 minutes prior to allergen challenge. Vehicle-treated mice received PBS.

### Immunofluorescence staining.

Immunofluorescence staining was performed as previously reported (71). Antibody information is provided in Supplemental Table 3. Fluorescence signals in tissue sections were quantified using ImageJ v1.50e (NIH) in 4 different high-power fields from each lung section and presented as mean fluorescence intensity per square micrometer.

### Western blotting.

Tissues or cells were lysed with RIPA buffer containing protease and phosphatase inhibitor cocktails (Sigma-Aldrich). Protein concentrations were measured by using BCA kit (Thermo Fisher Scientific). A total of 30 μg of lysed protein was separated by SDS-PAGE and then transferred onto a nitrocellulose membrane. After blocking with 5% no-fat milk in TBST for 1 hour at room temperature, the membranes were incubated with primary antibodies overnight at 4°C (Supplemental Table 1). Membranes were washed and probed with IRDye 800CW– or IRDye 680RD–conjugated secondary antibody (LI-COR Biosciences) for 1 hour at room temperature. Detection was performed using a LI-COR Odyssey CLx imaging system, and fluorescence intensity was quantified using Image Studio Lite version 5.2.5 (LI-COR Biosciences).

### Histological analysis.

Lungs were perfused and fixed with 4% formalin, embedded in paraffin, and then sectioned at 4 μm. After deparaffinization and dehydration, the sections were subjected to H&E and PAS staining to evaluate general morphology and mucus production as previously described (71).

### Flow cytometry.

Single-cell suspensions were prepared from BALF or lung tissue by mechanical disruption without digestive enzymes. Cellular differential percentages were measured by flow cytometry on an Accuri C6 Plus Flow Cytometer (BD Biosystems), and the data were analyzed with FlowJo software (Tree Star Inc.). A list of antibodies used for flow cytometry is presented in Supplemental Table 1. Eosinophils were defined as SSC^hi^SiglecF^+^Mac3^–^, alveolar macrophages as SSC^hi^SiglecF^+^Mac3^+^, granulocytes as SSC^hi^Gr-1^+^, and lymphocytes as FSC^lo^SSC^lo^CD3^+^ cells (72). Tregs were characterized as CD4^+^CD25^+^ cells expressing the intracellular FoxP3 transcriptional factor (73). ILC2s were defined as lineage^–^ (cells not expressing CD3, CD4, CD5, CD8α, CD11b, CD11c, CD19, B220, NK1.1, FcεRIα, TER-119, and Gr-1) CD45^+^CD127^+^ ST2^+^cells (74).

### ELISA.

Levels of cytokines in cell-free BALF or supernatant were quantified by using the Ready-Set-Go! ELISA sets (Thermo Fisher Scientific). CRE-specific IgE and IgG1 serum levels were analyzed by ELISA as previously described (71). Human SLA26A4 in serum was detected by using Human SLC26A4 ELISA kit (orb562738, Biorbyt).

### RNA isolation and qPCR analysis.

Total RNA was isolated using the Monarch Total RNA Miniprep Kit (NEB), and cDNAs were synthesized with the High-Capacity cDNA Reverse Transcription Kit (Thermo Fisher Scientific) as instructed by the manufactures. Real-time PCR was performed using Power SYBR Green PCR Master Mix (Thermo Fisher Scientific) on an ABI Prism 7300 detection system. Data were analyzed using the 2^–ΔΔCT^ method relative to the housekeeping gene GAPDH (75). Primer sequences are available in Supplemental Table 4.

### Isolation of mouse AT2 cells.

Mouse AT2 cells were isolated as previously reported (76, 77). Briefly, mouse was euthanized with ketamine and xylazine intraperitoneally. Mice were perfused with DPBS (Thermo Fisher Scientific) and then injected with 1.5–2 mL of dispase (Thermo Fisher Scientific) and 0.5 mL of 1% low-melting agarose (Thermo Fisher Scientific) into the lung via trachea. After removing trachea and airways, lung tissues were incubated with AT2 isolation medium consisting of a 1:1 mixture of DMEM and Ham’s F-12 (DMEM/F-12; MilliporeSigma) supplemented with 0.01% DNase I (MilliporeSigma), 100 U/mL sodium penicillin G (Thermo Fisher Scientific), and 100 μg/mL streptomycin (Thermo Fisher Scientific). The mixture was filtered through cell strainers and then resuspended with AT2 isolation medium containing a 1:1 mixture of DMEM/F-12 supplemented with 10% FBS (Thermo Fisher Scientific), 100 U/mL sodium penicillin G, and 100 μg/mL streptomycin. Cells were stained with biotinylated antibodies (including anti-CD16/32, anti-CD45, anti-Ter119, and anti–Sca-1, BioLegend; Supplemental Table 1) at 37°C shaking incubator for 30 minutes and subsequently subjected to magnetic selection with streptavidin-conjugated magnetic beads for 30 minutes at room temperature. Cells were centrifuged for 10 minutes at 300*g* at 4°C, and they were incubated on precoated dishes with mouse IgG. After incubation for 2 hours, nonadherent cells were collected, centrifuged (300*g* for 5 minutes at 4°C), and resuspended with AT2 isolation medium.

### RNA-seq analysis.

RNA-seq libraries were prepared for sequencing using Illumina TruSeq stranded mRNA sample preparation kit following the manufacturer’s recommended procedure. Briefly, total RNA was extracted using TRIzol Reagent. mRNA was enriched using oligo(dT) beads and then fragmented chemically by incubating at 94°C for 8 minutes. cDNA was synthesized with SuperScript II. After purification using AMPure XP beads, the double-stranded cDNA was ligated to TruSeq RNA adapters followed by 15 cycles of amplification and library purification. Sequencing was performed on an Illumina NextSeq500. RNAseq reads were aligned to the mouse reference genome GRCm38 using STAR aligner version 2.7.2b. BAM file outputs from STAR were annotated using Partek Genomic Suite (v6.6) and the RefSeq database (*RefSeq 21*). MIAME-compliant gene expression data have been deposited to the NCBI’s Gene Expression Omnibus (GEO; GSE175932) (78). Differential analysis was performed with Cufflink 2.21. Fold induction of gene expression (average ± SEM) was calculated from RNA expression in CRE-treated WT group when compared with CRE-treated RhoA-KO group.

### Cell culture and transfection.

HBECs (ATCC, CRL-4051) were cultured in Ham’s F-12K (Kaighn’s) medium supplemented with 10% v/v FBS and 1% penicillin-streptomycin. The transfection of DNA plasmids was performed with FuGENE 6 Transfection Reagent (Promega). The transfection of siRNA was performed with Lipofectamine RNAiMAX Reagent (Thermo Fisher Scientific).

### Human lung tissues.

We used the existing Paraffin-embedded human airway sections from asthmatic and healthy individuals stored at the Johns Hopkins Asthma Center Histology laboratory.

### Human serum samples.

Serum samples from asthmatic patients and healthy subjects were collected from Guangzhou Institute of Respiratory Health, State Key Laboratory of Respiratory Disease (Guangzhou Medical University). Asthma was diagnosed according to the Global Initiative for Asthma (GINA) 2019 (79). The severity of disease for each patient was based on GINA 2018/National Asthma Education and Prevention Program guidelines. Healthy controls had no symptoms of chronic cough or wheeze in the last 12 months and no history of chronic lung diseases (e.g., asthma, COPD, cystic fibrosis, bronchiectasis, lung cancer) with normal lung function. All the detailed clinical and demographic data for these study subjects are described in the Supplemental Methods and Supplemental Table 1.

### Statistics.

All data are presented as mean ± SEM for each group and analyzed with GraphPad Prism version 8.1 software (GraphPad Software). Statistical significance was assessed using an independent 2-tailed Student’s *t* test for comparisons between 2 groups or with 2-way ANOVA for comparisons among multiple groups. For the correlation analysis, data were firstly tested for normal distribution using the Kolmogorov-Smirnov test and then analyzed for correlation using Pearson Product-Moment Correlation. A *P* value of less than 0.05 was considered statistically significant.

### Study approval.

The animal care and experiments were performed in compliance with the institutional and US NIH guidelines and were reviewed and approved by the Johns Hopkins University Animal Care and Use Committee. The existing paraffin-embedded human airway sections from asthmatic and healthy individuals for this study was approved by the Johns Hopkins IRB with an approval no. NA_00046988. Serum samples from asthmatic patients and healthy subjects were collected from Guangzhou Institute of Respiratory Health, State Key Laboratory of Respiratory Disease (Guangzhou Medical University). The study was approved by the ethics committee of the First Affiliated Hospital of Guangzhou Medical University (YKLS-201718). All the patients and healthy donors consented through written and informed agreement for inclusion in the study.

## Author contributions

DCD, YZ, WT, XH, XX, JC, and HH performed experiments and analyzed data. DCD and PG wrote the manuscript. JL provided clinical samples. ZL, SKH, MW, and PG designed and supervised the study and revised the manuscript. All authors read and approved the final manuscript.

## Supplementary Material

Supplemental data

## Version 1 06/08/2021

In-Press Preview

## Version 2 07/22/2021

Electronic publication
