# Supplementary material for: Type II alveolar epithelial cell–specific loss of RhoA exacerbates allergic airway inflammation through SLC26A4
Source: JCI Insight. 2021 Jul 22;6(14):e148147. doi: 10.1172/jci.insight.148147 (PMC8410088; doi:10.1172/jci.insight.148147)
Supplement: Supplemental data [file jciinsight-6-148147-s152.pdf]

Supplementary Materials

Type II Alveolar Epithelial Cell–Specific Loss of RhoA Exacerbates Allergic Airway Inflammation through SLC26A4

Danh C Do[1*], Yan Zhang[1,2*], Wei Tu[1,3], Xinyue Hu[1,2], Xiaojun Xiao[4], Jingsi Chen[5], Haiping Hao[6], Zhigang Liu[3,4], Jing Li[7], Shau-Ku Huang[1,8], Mei Wan[9], Peisong Gao[1*]

[1]Division of Allergy and Clinical Immunology, Johns Hopkins University School of Medicine, Baltimore, MD 21224, USA

[2]Department of Respiratory Medicine, Xiangya Hospital, Central South University, Changsha, Hunan 410008, China

[3]Department of Respirology & Allergy, Third Affiliated Hospital of Shenzhen University, Shenzhen 518020, China.

[4]Institute of Allergy and Immunology, School of Medicine, Shenzhen University, Shenzhen, China.

[5]Children's Hospital, Chongqing Medical University, Chongqing 400014, China.

[6]JHMI Deep Sequencing and Microarray Core Facility, Johns Hopkins University School of Medicine, Baltimore, MD 21224, USA.

[7]Department of Allergy and Clinical Immunology, Guangzhou Institute of Respiratory Health, State Key Laboratory of Respiratory Disease, The First Affiliated Hospital of Guangzhou Medical University, Guangzhou, China.

[8]National Institute of Environmental Health Sciences, National Health Research Institutes, Taiwan

[9]Department of Orthopaedic Surgery, Johns Hopkins University School of Medicine, Baltimore, Maryland, USA

*These authors contributed equally to this work.

Materials and Methods

Measurement of airway hyper-responsiveness

Mice were anesthetized with a ketamine (90 mg/kg)/xylazine (18 mg/kg) mixture, and a tracheotomy tube was inserted. Ventilation was initiated with a volume-cycled ventilator (Flexivent; SCIREQ Scientific) with a positive-end expiratory pressure of 2 cmH2O). Airway responsiveness was monitored by challenging mice with a dose-dependent aerosolized methacholine (0-30 mg/ml). The airway resistance was measured with the Flexivent software and exported to Pulmodyn data-acquisition software (Hugo Sachs Electronic) for data analysis (1).

Human subjects

The healthy subjects had normal lung function and did not suffer from respiratory or other diseases. Asthma was diagnosed according to the Global Initiative for Asthma (GINA). The degree of severity was based on an individual's medication use, recommended by GINA (2). This study included 55 asthma patients and classified as mild-to-moderate for 31 patients (steps1-3) and severe for 24 patients (step 4-5). During the week before the study, all subjects did not take any drugs with steroid and some of them only take short-acting inhaled beta-2 agonists as needed. All participants were lifetime non-smokers, and had no evidence of COPD. We collected blood samples from patients when they came to the clinic, and collected the information on their socio-demographic status. Lung function and eosinophils and basophils in peripheral blood were evaluated. In all the 3 sets of human samples, both age and sex showed similar distribution in all the 3 sets of human samples.

Detection of Human serum SLC26A4 (Pendrin) by ELISA

This assay employs a two-site sandwich ELISA (orb562738, Biorbyt) to quantitate SLC26A4 in samples according to the manufacturer's instructions. An antibody specific forSLC26A4 has been pre-coated

onto a microplate. Standards and samples are pipetted into the wells and anySLC26A4 present is bound by the immobilized antibody. After removing any unbound substances, a biotin-conjugated antibody specific for SLC26A4 is added to the wells. After washing, Streptavidin conjugated Horseradish Peroxidase (HRP) is added to the wells. Following a wash to remove any unbound avidin-enzyme reagent, a substrate solution is added to the wells and color develops in proportion to the amount of SLC26A4 bound in the initial step. The color development is stopped and the intensity of the color is measured.

References

1. Zhang Y, Do DC, Hu X, Wang J, Zhao Y, Mishra S, et al. CaMKII oxidation regulates cockroach allergen-induced mitophagy in asthma. *The Journal of allergy and clinical immunology*. 2021;147(4):1464-77 e11.

2. Boulet LP, Reddel HK, Bateman E, Pedersen S, FitzGerald JM, and O'Byrne PM. The Global Initiative for Asthma (GINA): 25 years later. *The European respiratory journal*. 2019;54(2).

Figure legends

Fig. S1. Generation of type II alveolar epithelial RhoA deficient mice. (A) Schematic representation of the crossbreeding of a floxed RhoA mouse (RhoA$^{f/f}$) with a Sftpc-cre mouse. (**B-C**) Confirmation of Sftpc-cre:RhoA$^{f/f}$ mice by genotyping (**B**) and co-immunostaining with Sftpc and RhoA in the lung tissues (**C**). (**D**) Representative immunofluorescence images of Sftpc expression in AT1, central airways, and basal cells of Sftpc-cre; RhoAf/f mice by co-immunostaining Sftpc with Pdpn, cc10, and Krt5, respectively. (**E**) Representative immunofluorescence images of RhoA expression in AT1, central airways, and basal cells of RhoA$^{f/f}$ and Sftpc-cre; RhoAf/f mice by co-immunostaining RhoA with Pdpn, cc10, and Krt5, respectively.

Fig. S2. Deletion of RhoA in AT2 cells leads to the exacerbation of CRE-induced airway hyper-responsiveness. Lung resistance in response to increasing concentrations of methacholine using the forced oscillation technique (FlexiVent, SCIREQ). (n=8). Data represent mean ± SEM of two independent experiments. $^{**}P < 0.01$, $^{***}P < 0.001$.

Fig. S3. Number of neutrophils in the BAL fluids of mouse model of asthma. (**A-D**) Number of neutrophils in the BAL fluids of cockroach allergen (CRE, n=8, **A**), house dust mite (HDM, n=7, **B**), rTgf-β1-treated (n=7, **C**), and NPPB-treated (n=10, **D**) mouse models of asthma. Data represent mean ± SEM of two independent experiments. Group comparisons were made using 2-way ANOVA. $^{*}P < 0.05$, $^{***}P < 0.001$.

Fig. S4. Deletion of RhoA in AT2 cells leads to the exacerbation of house dust mite (HDM)-induced airway inflammation. (**A**) Protocol for HDM-induced mouse model of asthma. **(B)** Histological examination of mouse paraffin lung sections stained with hematoxylin and eosin (H&E, upper panel) and Periodic acid–Schiff (PAS, lower panel). (**C**) Quantification of mononuclear cell infiltrates in H&E stained lung sections (n=7). (**D**) Goblet cells quantification in PAS-stained lung sections (n=7). (**E**) Bronchoalveolar lavage (BAL) fluid total and eosinophil cell counts as determined

by flow cytometry (n=7). (**F**) Serum levels of cockroach allergen-specific IgE and IgG1 (n=7). (**G**) BAL fluid levels of cytokines (n=7). Data represent mean ± SEM of two independent experiments. Group comparisons were made using 2-way ANOVA. **P* <0.05, ***P* <0.01, and ****P* <0.001.

Fig. S5. Detection of Th1, Th2, and Th17 cells in lung tissues as assessed by flow cytometry. (A) Gate strategies for Th1, Th2, and Th17 detection. (**B**) Numbers of Th1, Th2, and Th17 (n=6-8). Data represent mean ± SEM of two independent experiments. Group comparisons were made using 2-way ANOVA. **P* <0.05, ***P* <0.01, and ****P* <0.001.

Fig. S6. SLC26A4 knockdown in HBECs. Western blot confirmation of SLC26A4 knockdown in HBECs by SLC26A4 siRNA.

Table S1. Characteristics of human subjects

	**Healthy controls (n=18)**	**Mild Asthma (n=31)**	**Severe Asthma (n=24)**	***p-value***
Age (Year)	38 (28-48)	39 (32-52)	49 (36-54)	0.236
Gender (Female, %)	10 (55.56)	17 (54.84)	16 (66.66)	0.569
Body Mass Index	18.63 (15.46-23.91)	18.32 (15.27-27.04)	18.11 (14.41-23.81)	0.508
Age of Asthma Onset (Year)	NA	33 (19-48)	32 (26-43)	0.823
ACT score	NA	20 (18-23)	17 (12-22)	0.034
FeNO (ppb)	NA	63.0 (37.5-126.0)	58.0 (25.0-69.0)	0.317
Parameters of Lung Function				
FEV_1 (% predicted)	NA	83.43 (71.42-93.15)	48.30 (37.05-58.32)	<0.001
FEF_{25-75} (% predicted)	NA	43.40 (31.12-55.97)	14.50 (11.70-20.86)	<0.001
FVC (% predicted)	NA	102.55 (86.92-114.92)	77.80 (72.40-86.75)	<0.001
FEV_1/FVC	NA	77.89 (71.99-86.12)	52.20 (47.07-68.85)	<0.001
Peripheral Eosinophil Count (10^9/L)	0.36 (0.24-0.49)	0.32 (0.21-0.44)	0.45 (0.35-0.71)	0.022
Allergen Sensitizations (%)	NA	23.00 (71.88)	10.00 (47.61)	<0.001
Serum Total IgE (IU/mL)	NA	260.0 (107.0-378.0)	225.5 (82.10-490.0)	0.950

NA, Not Applicable; MMA, Mild or Moderate Asthma; SA, Severe Asthma. *P* value was calculated using the $\chi 2$ test for categorical variables and ANOVA for continuous variables.

Table S2. Correlations between serum levels of pendrin and pulmonary function parameters among asthmatic patients

SUBGROUP (NUMBER)	PARAMETER	R (95% CI)	P VALUE
**MMA AND SA (N=53)**			
	·FEV_1(% predicted)	-0.2734	**0.0476***
	·FEF_{25-75}(% predicted)	-0.1966	0.1582
	·FVC(% predicted)	-0.2224	0.1095
	·FEV_1/FVC	-0.1991	0.1529
**MMA (N=30)**			
	·FEV_1 (% predicted)	-0.4674	**0.0092****
	·FEF_{25-75} (% predicted)	-0.3352	0.0701
	·FVC (% predicted)	-0.3474	0.0600
	·FEV_1/FVC	-0.2648	0.1573
**SA (N=23)**			
	·FEV_1(% predicted)	-0.1989	0.3628
	·FEV_1/FVC	-0.1256	0.5679

MMA, Mild or Moderate Asthma; SA, Severe Asthma. *P<0.05, **P<0.01.

Table S3: Antibodies used for flow cytometry, western blot, and immunofluorescence

Target	Specie	Clone	Assay	Company
CD25	anti-Ms	PC61.5	FC (0.5 µg/mL)	BioLengend
CD4	anti-Ms	RM4-5	FC (0.5 µg/mL)	BioLengend
CD45	anti-Ms	30-F11	FC (0.5 µg/mL)	BioLengend
CD127	anti-Ms	A7R34	FC (0.5 µg/mL)	BioLengend
FoxP3	anti-Ms	150D	FC (1.0 µg/mL)	BioLengend
Lineage (CD3, CD4, CD11b, CD11c, B220, NK1.1, FcεRIα, TER-119, and Gr-1)	anti-Ms	145-2C11, RM4-4, M1/70, N148, RA3-6B2, PK136, MAR-1, TER-119, RB6-8C5	FC (1:50)	BioLengend
RhoA	anti-Hu, Ms	10749-1-AP	IF (1:250)<br>WB (1:1000)	PTGLAB
RhoA-GTP	anti-Hu	26904	WB (1:200)	NewEast
SFTPC	anti-Hu	4A10	IF (1:50)	Abnova
SLC26A4	anti-Hu, Ms	NBP1-60106	IF (20 µg/mL)	Novus
CC10	anti-Hu, Ms	10490-AP	IF (1:250)	Proteintech
Krt5	anti-Hu, Ms	Bs-5352R	IF (1:100)	Bioss Antibodies
Pdpn	anti-Ms	8.1.1	IF (1:200)	BioLegend
ST2	anti-Ms	D1H9	FC (0.5 µg/mL)	BioLengend
Tgf-β1	anti-Ms	NBP1-45891	IF (1:100)	Novus
β-actin	anti-Ms	C-2	WB (1:5000)	SCBT
Rb IgG1 isotype	-	DA1E	IF (1:100)	Cell Signaling
Ms IgG1 isotype	-	G3A1	IF (1:50)	Cell Signaling

Rb: rabbit, Ms: mouse, Hu: human, FC: Flow cytometry, WB: Western Blot, and IF: Immunofluorescence

Table S4: Primer sequences for RT-qPCR

Gene	NCBI Gene ID	Primer Bank ID	Amplicon Size	Sequence
Slc26a4	23985	6755022a1	147	F: 5'AAGAGAGCCTTTGGTGTGGTA<br>R: 5'CAGGGCATAAGCCATCCCTTG
Retnla	57262	10048446a1	108	F: 5'CCAATCCAGCTAACTATCCCTCC<br>R: 5'ACCCAGTAGCAGTCATCCCA
Slc34a2	20531	6755556a1	130	F: 5'CCTTGGCCCGAGTTGGAAAAT<br>R: 5'CTACAGGAGTCCCGTTGTCAT
Egfl8	81701	23463330a1	104	F: 5'TGGGCTGAGCTGTGCATTT<br>R: 5'CACACTCCCAAACTCTCTTTGAA
Actb	11461	6671509a1	154	F: 5'GGCTGTATTCCCCTCCATCG<br>R: 5'CCAGTTGGTAACAATGCCATGT
SLC26A4	5172	4505696c1	211	F: 5'GCTCCCCAAATACCGAGTCAA<br>R: 5'CACCACTGGAAAAGGTCCAAC
GAPDH	2597	378404907c1	197	F: 5'GGAGCGAGATCCCTCCAAAAT<br>R: 5'GGCTGTTGTCATACTTCTCATGG

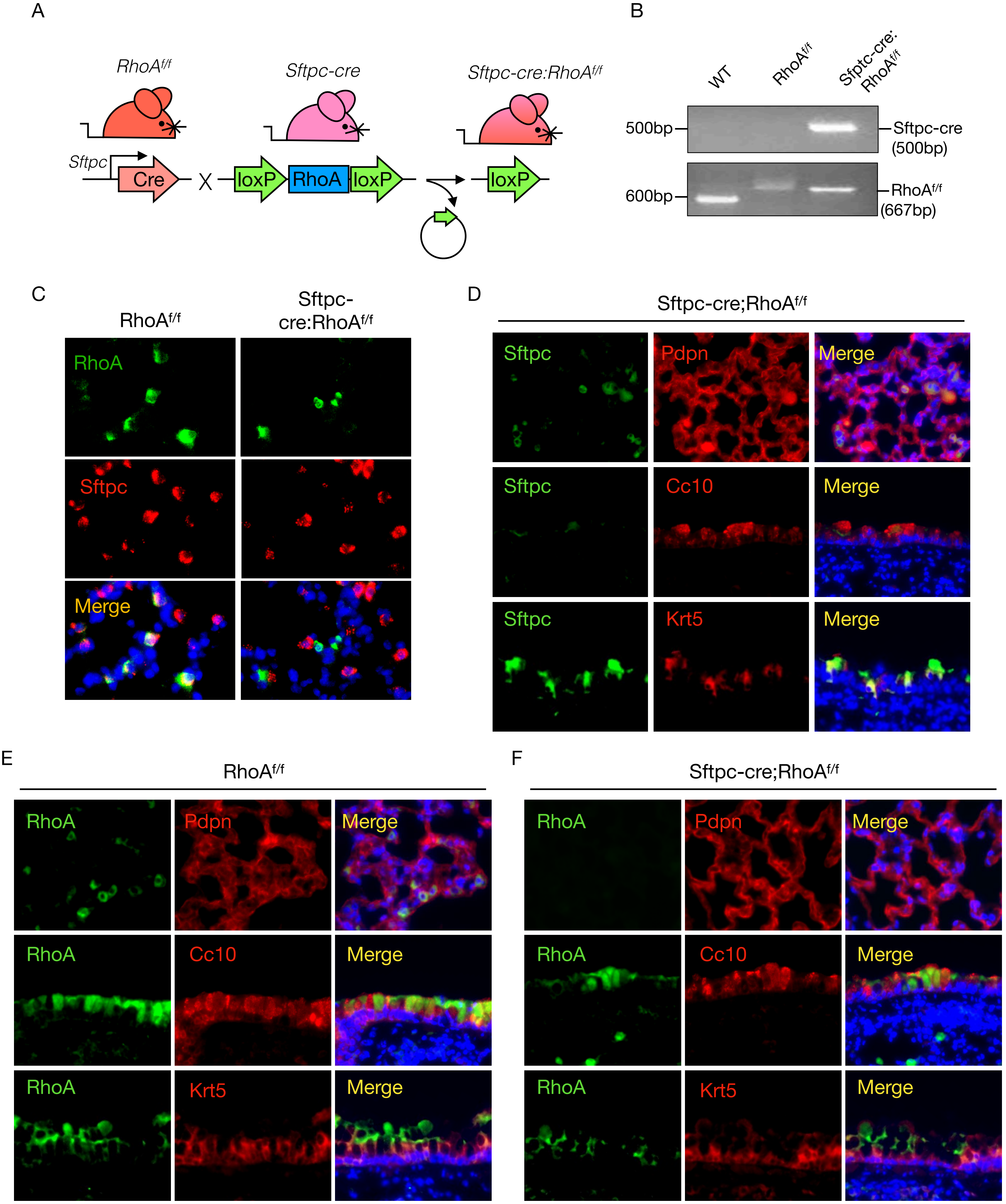
A
RhoAf/f
Sftpc-cre
Sftpc-cre:RhoAf/f
Sftpc
Cre
X
loxP
RhoA
loxP
loxP
B
WT
RhoAf/f
Sfptc-cre:
RhoAf/f
500bp
600bp
Sftpc-cre
(500bp)
RhoAf/f
(667bp)
C
RhoAf/f
Sftpc-
cre:RhoAf/f
RhoA
Sftpc
Merge
D
Sftpc-cre;RhoAf/f
Sftpc
Pdpn
Merge
Sftpc
Cc10
Merge
Sftpc
Krt5
Merge
E
RhoAf/f
RhoA
Pdpn
Merge
RhoA
Cc10
Merge
RhoA
Krt5
Merge
F
Sftpc-cre;RhoAf/f
RhoA
Pdpn
Merge
RhoA
Cc10
Merge
RhoA
Krt5
Merge

Fig. S2

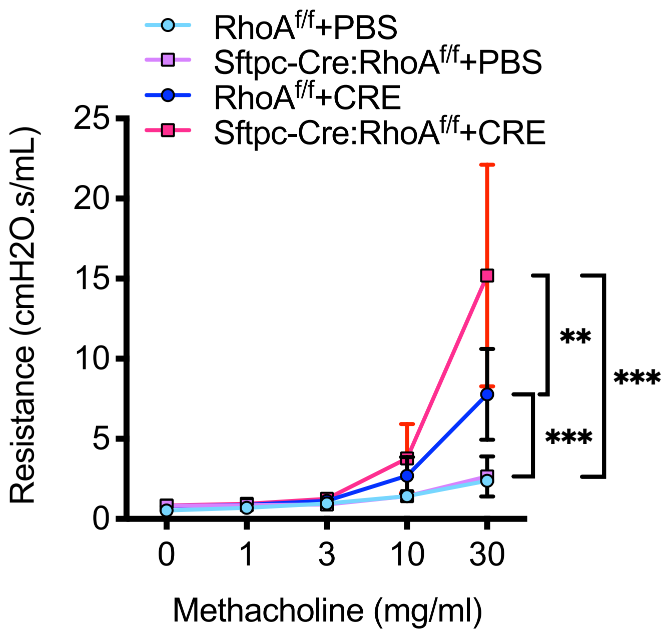

RhoA f/f+PBS
Sftpc-Cre:RhoA f/f+PBS
RhoA f/f+CRE
Sftpc-Cre:RhoA f/f+CRE
Resistance (cmH2O.s/mL)
25
20
15
10
5
0
0
1
3
10
30
Methacholine (mg/ml)
**
***
***

Fig. S3

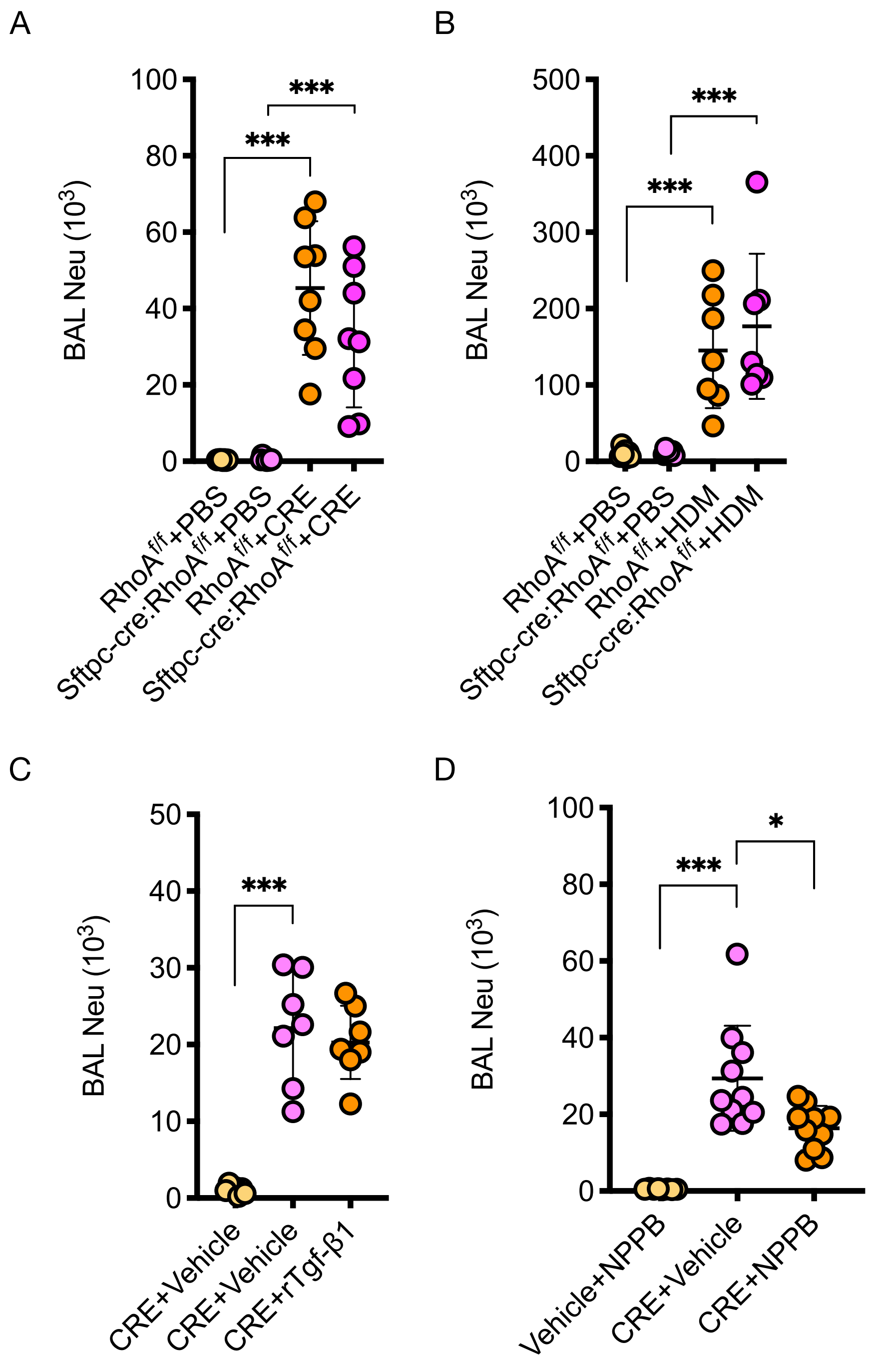
A
B
C
D
BAL Neu (10^3)
***
*
RhoA f/f+PBS
Sftpc-cre:RhoA f/f+PBS
RhoA f/f+CRE
Sftpc-cre:RhoA f/f+CRE
RhoA f/f+HDM
Sftpc-cre:RhoA f/f+HDM
CRE+Vehicle
CRE+rTgf-β1
Vehicle+NPPB
CRE+NPPB

Fig. S4

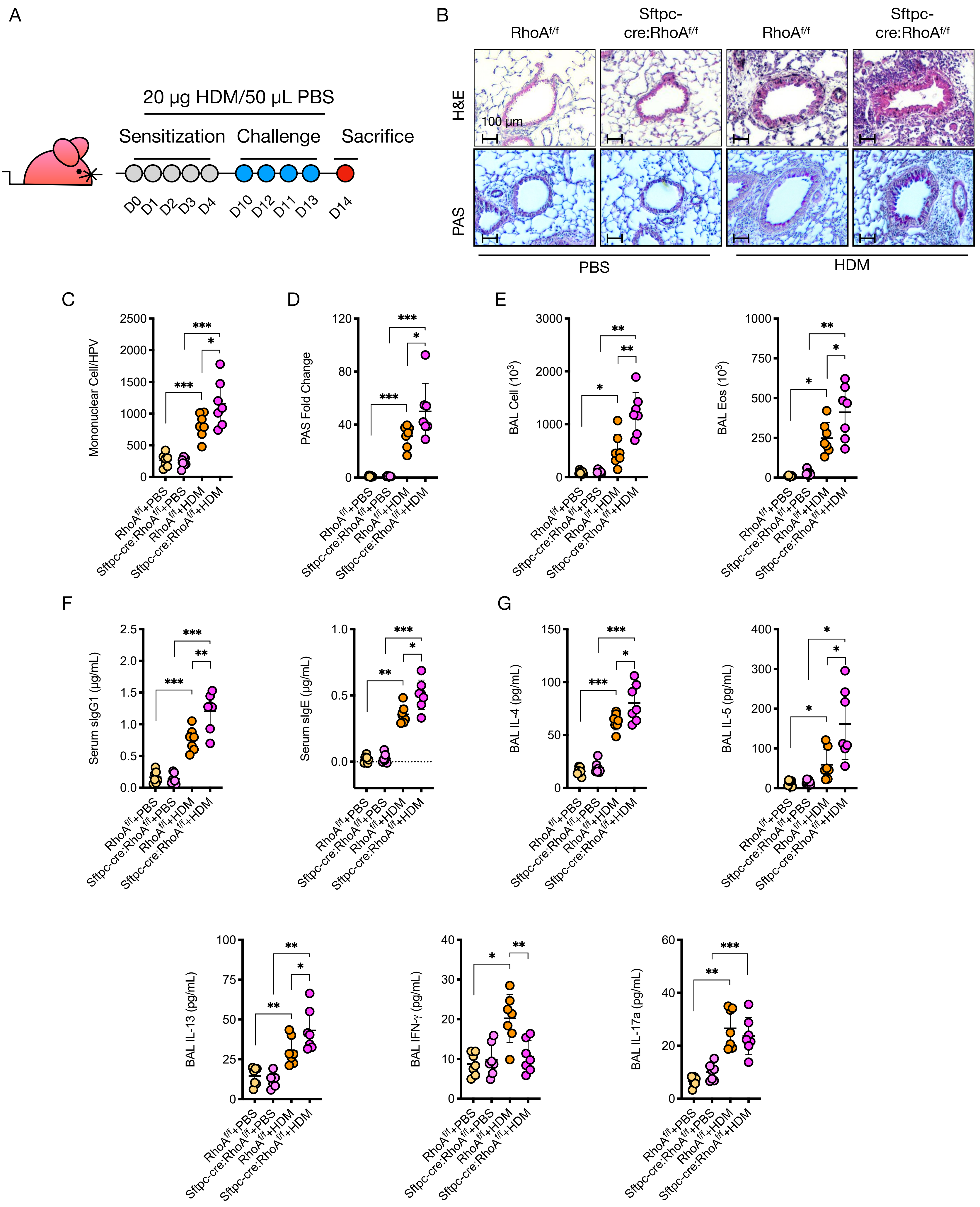

A
20 μg HDM/50 μL PBS
Sensitization
Challenge
Sacrifice
D0 D1 D2 D3 D4 D10 D12 D11 D13 D14
B
RhoA f/f
Sftpc-cre:RhoA f/f
H&E
PAS
100 μm
PBS
HDM
C
Mononuclear Cell/HPV
D
PAS Fold Change
E
BAL Cell (10^3)
BAL Eos (10^3)
F
Serum sIgG1 (μg/mL)
Serum sIgE (μg/mL)
G
BAL IL-4 (pg/mL)
BAL IL-5 (pg/mL)
BAL IL-13 (pg/mL)
BAL IFN-γ (pg/mL)
BAL IL-17a (pg/mL)
RhoA f/f+PBS
Sftpc-cre:RhoA f/f+PBS
RhoA f/f+HDM
Sftpc-cre:RhoA f/f+HDM

Fig. S5

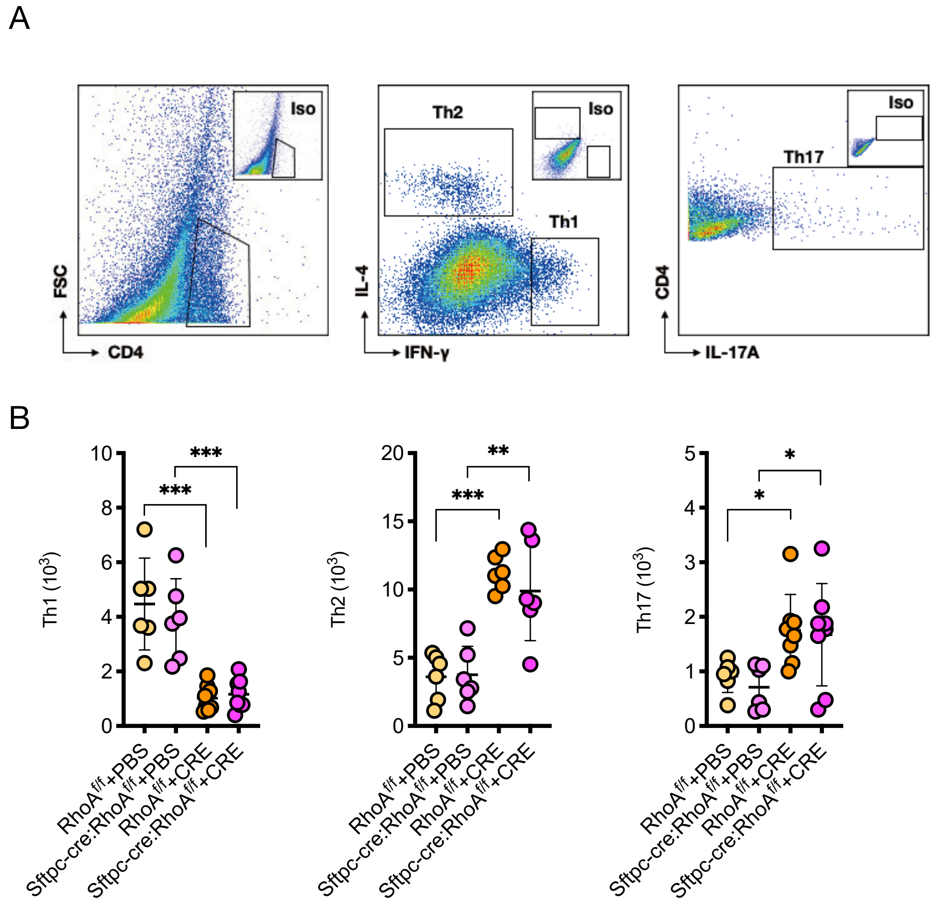
A
Iso
FSC
CD4
Th2
Th1
IL-4
IFN-γ
Th17
IL-17A
B
Th1 (10^3)
Th2 (10^3)
Th17 (10^3)
***
**
*
RhoA f/f+PBS
Sftpc-cre:RhoA f/f+PBS
RhoA f/f+CRE
Sftpc-cre:RhoA f/f+CRE

Fig. S6

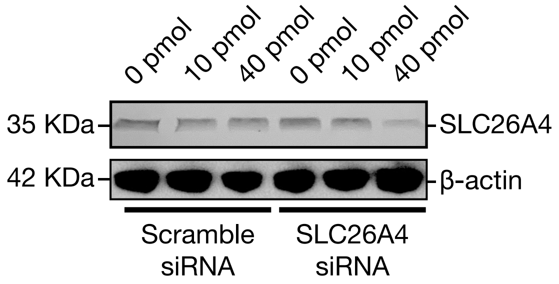
0 pmol
10 pmol
40 pmol
0 pmol
10 pmol
40 pmol
35 KDa
42 KDa
SLC26A4
β-actin
Scramble siRNA
SLC26A4 siRNA
